# Low‐Energy, Ultrafast Spin Reorientation at Competing Hybrid Interfaces with Tunable Operating Temperature

**DOI:** 10.1002/adma.202419192

**Published:** 2025-08-05

**Authors:** Servet Ozdemir, Matthew Rogers, Jaka Strohsack, Hari Babu Vasili, Manuel Valvidares, Thahabh Haddadi, Parvathy Harikumar, David O'Regan, Gilberto Teobaldi, Timothy Moorsom, Mannan Ali, Gavin Burnell, B J Hickey, Tomaz Mertelj, Oscar Cespedes

**Affiliations:** ^1^ School of Physics and Astronomy University of Leeds Leeds LS2 9JT UK; ^2^ Department of Complex Matter Jozef Stefan Institute and Center of Excellence on Nanoscience and Nanotechnology Nanocenter (CENN Nanocenter) Jamova 39 Ljubljana 1000 Slovenia; ^3^ ALBA Synchrotron Light Source Cerdanyol del Valles Barcelona E‐08290 Spain; ^4^ School of Physics and CRANN Institute Trinity College Dublin, The University of Dublin Dublin D02 PN40 Ireland; ^5^ Scientific Computing Department STFC UKRI Rutherford Appleton Laboratory Didcot OX11 0QX UK; ^6^ School of Chemical and Process Engineering University of Leeds Leeds LS2 9JT UK

**Keywords:** current induced switching, magnetism, phase transition, spin‐reorientation transition, ultrafast switching

## Abstract

Information can be stored in magnetic materials by encoding with the direction of the magnetic moment. A figure of merit for these systems is the energy needed to rewrite the information by changing the magnetic moment. Organic molecules offer a playground to manipulate spin order, with metallo‐molecular interfaces being a promising direction for sustainable devices. Here, a spin reorientation transition is demonstrated in molecular interfaces of 3d ferromagnetic films due to a competition between a perpendicular magnetic anisotropy (PMA) induced by a heavy metal that dominates at high temperatures, and an in‐plane anisotropy generated by molecular coupling at low temperatures. The transition can be tuned around room temperature by varying the ferromagnet thickness (1.4 – 1.9 nm) or the choice of molecular overlayer, with the organic molecules being C_60_, hydrogen, and metal (Cu, Co) phthalocyanines. Near the transition temperature, the magnetisation easy axis can be switched with a small energy input, either electrically with a current density of 10^5^ **A** **cm** ^−2^, or optically by a fs laser pulse of fluence as low as 0.12 **mJ** **cm** ^−2^, suggesting heat assisted technology applications. Magnetic dichroism measurements point toward a phase transition at the organic interface being responsible for the spin reorientation transition.

## Graphical abstract

A spin reorientation transition is generated at hybrid interfaces of Pt‐Co‐organic molecules in the vicinity of room temperature. This spin reorientation transition happens at an ultrafast scale and is controlled either through electrical current or fs laser pulse, introducing perpendicular magnetic anisotropy in formerly planar magnetised samples through heating.

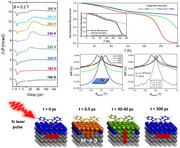

## 1 Introduction

Conventional spin reorientation transitions take place when the easy axis of magnetisation rotates with respect to the crystallographic axes due to changes in temperature, pressure, or magnetic field. The effect has been widely studied since the 1940s^[^ ^1^ ^]^ for both rare earth systems^[^ ^2^ ^]^ and magnetic semiconductors.^[^ ^3^ ^]^ The exponential increase of digital content generated and exchanged as we approach the yottabyte era, combined with environmental objectives, has seen information technologies focus on memory and storage devices made with sustainable materials that allow increased density, lower power consumption, and reduced cooling requirements.^[^ ^4^ ^]^ The search is hampered by the relatively large energies and long times needed to either rotate the magnetisation in conventional hard disks and STT/SOT memories^[^ ^5^, ^6^ ^]^ or to erase it in heat‐assisted storage devices.^[^ ^7^ ^]^ Hence, the incorporation of new mechanisms such as spin reorientation transitions into these technologies is an attractive possibility to reduce power consumption. Dilute magnetic semiconductors have been suggested as contenders where a spin reorientation transition takes place, but low operational temperatures and small magnetisation hamper their application.^[^ ^3^, ^8^ ^]^ Switching that depends on crystallographic axes requires high crystal quality that is difficult to reproduce in commercial nanodevices. Therefore, finding a system where the magnetisation easy axis can be switched within a narrow, controlled temperature range and independently of the crystal structure would be highly beneficial. Interfacial spin dynamics of common transition metals with cheap, eco‐friendly organic molecules give rise to emergent functionalities ideal for the observation of novel magnetic phenomena such as a large interfacial tunnel magnetoresistance,^[^ ^9^ ^]^ ferromagnetism onset,^[^ ^10^ ^]^ as well as magnetic hardening.^[^ ^11^, ^12^ ^]^ Here, we report on metallo‐molecular interfaces with an easy axis switching, or spin reorientation transition^[^ ^1^, ^2^ ^]^ around room temperature with low‐energy electrical or optical inputs that lead to high magnetisation changes in ps timescales. The spin reorientation transition temperature, *T_S_*, is controlled through the ferromagnet thickness as well as the molecule used, enabling tunability of the operational temperature as well as device compatibility with standard room temperature information storage and computing technologies.

## 2 Results and Discussion

### 2.1 Universal Magnetic Hardening and Spin‐Reorientation Transition at Magneto‐Molecular Interfaces

**Figure 1:**
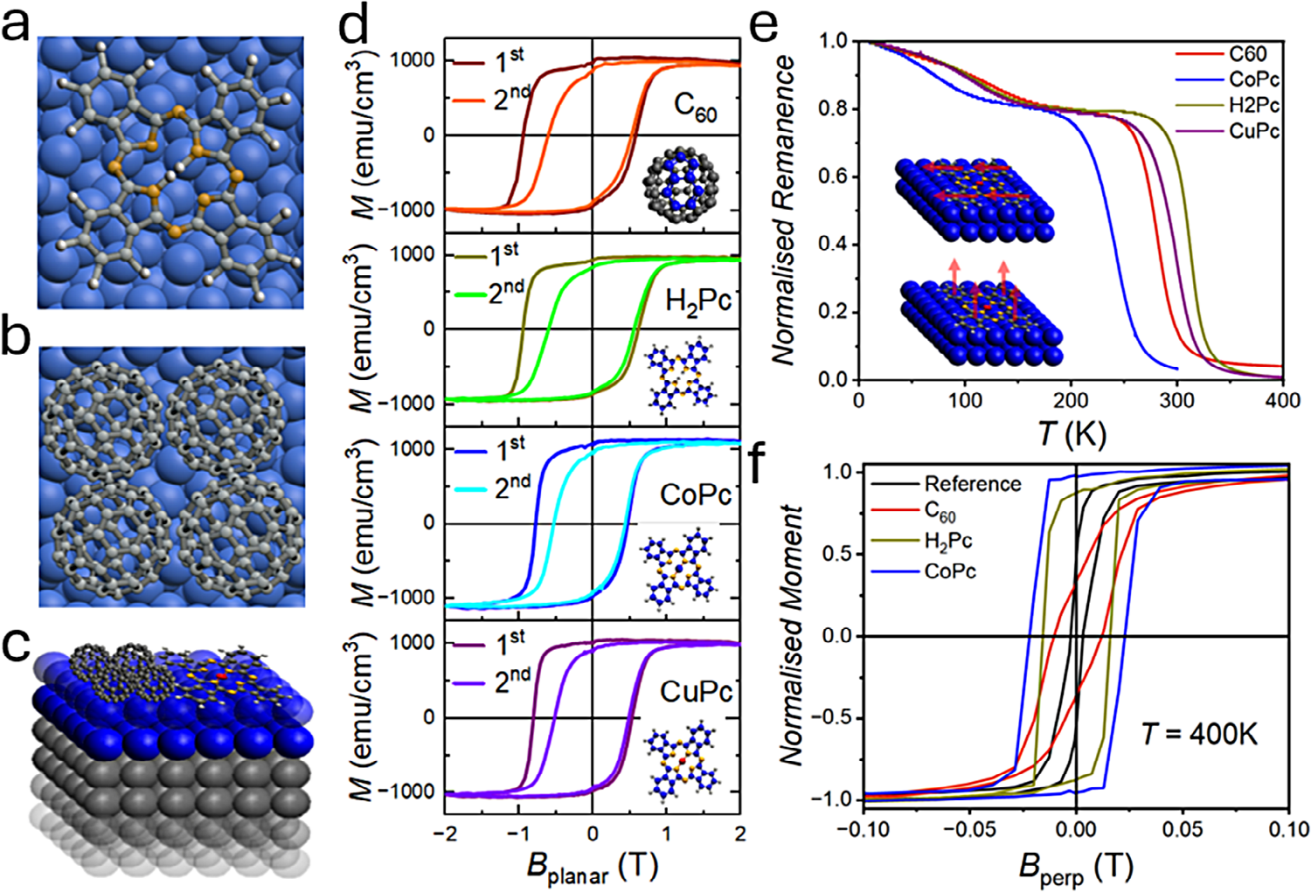
Magnetic hardening and spin reorientation transition at metallo‐molecular thin film interfaces. a) A H_2_‐Pc molecule (diameter ≈1.4 nm) lying in a planar orientation on a Co surface. b) Four C_60_ molecules (diameter ≈0.7 nm) hybridised with Co surface on hexagon‐pentagon faces occupying the same area as a single Pc molecule. c) Thin film structure depicted with the Pt seed layer (gray), Co‐layer (blue) with metal Pc and C_60_ molecules on the Co surface. d) In‐plane magnetisation loops (1st and 2nd loops depicted in different colors) of ≈1.7 nm Co thickness Pt/Co/C_60_, Pt/Co/H_2_Pc, Pt/Co/CoPc and Pt/Co/CuPc film structures measured at T = 10 K post field cooling at 2 T (corresponding molecules to each curve shown on the insets). e) Normalised in‐plane magnetic remanence measured post field cooling at 2 T for films of C_60_, H_2_Pc, CoPc, and CuPc interfaced with Pt/Co(1.7 nm) structure with schematics on the inset depicting the spin reorientation transition. f, Normalised perpendicular to plane hysteresis loops measured at T = 400 K for reference Pt/Co(1.7 nm) films as well as Pt/Co/C_60_, H_2_Pc and CoPc interfaces showing an enhancement in coercivity.

A comparative study involving molecules of different geometric and magnetic properties is needed to understand the magnetic interactions at the interface,^[^ ^13^, ^14^, ^15^ ^]^ see **Figure** **1**a–c. Although different crystal structures are possible, Phthalocyanine (Pc) molecules have been found to grow as α‐phase in a flat lying orientation at the interface on various substrates,^[^ ^16^ ^]^ particularly on (111) metal surfaces.^[^ ^17^, ^18^, ^19^ ^]^ C_60_ and H_2_Pc are diamagnetic, whereas metal phthalocyanines (CuPc, CoPc) have shown magnetic order at low temperatures.^[^ ^20^, ^21^, ^22^ ^]^ We find that, despite their varying intrinsic properties, Pt(111)/Co(*t* ≤ 2 nm)/molecular systems exhibit an in‐plane magnetisation accompanied by magnetic hardening and very large coercivities of the order of 1 T at low temperatures (Figure 1d). By contrast, reference Pt(111)/Co(*t* ≤ 2 nm) samples without competing molecular interfaces show PMA with an out‐of‐plane (OOP) easy axis at all temperatures (Figure S1, Supporting Information).^[^ ^23^ ^]^ The large coercivity is thought to be due to hybridisation and spin polarised charge transfer to the molecular interface (also known as a “spinterface”)^[^ ^24^ ^]^ which induces pinning^[^ ^15^ ^]^ of the magnetic moment of Co. After the first magnetisation reversal at low temperatures, the coercivity of all samples with a molecular interface reduces to 0.5–0.6 T, which is still one to two orders of magnitude larger than in plain Co films, confirming magnetic hardening of the hybridised Co layer.^[^ ^12^ ^]^ All four molecular interfaces share this behavior, suggesting that the origin of the magnetic hardening (the large coercivity) is the same for all samples. Additionally, the influence of central metal ions of CoPc and CuPc molecules can be ruled out to be at the origin of the hardening, because H_2_Pc and C_60_ molecules do not have these central metal ions. Equally, the spherical geometry of C_60_^[^ ^15^ ^]^ can be ruled out because phthalocyanine molecules are flat. There have been other cross‐molecule studies at Co interfaces where identical spin‐polarisation effects at the interface have been observed.^[^ ^25^ ^]^ The common feature between all molecules used in this study is the hexagonal‐pentagonal carbon unit. Density functional theory (DFT) calculations for Co/C_60_ interfaces have shown C_60_ molecules to hybridise with Co atoms in an orientation involving both hexagonal‐pentagonal faces.^[^ ^12^, ^15^ ^]^ Furthermore, studies on H_2_Pc metal interfaces have shown the spin polarisation effects focused on hexagonal‐pentagonal units.^[^ ^26^ ^]^ Remarkably, a simple geometrical comparison of fullerenes and Pcs leads one to find that 4 hexagon‐pentagon units are present in the same area of ≈1.54 nm^2^ for both, enabling d‐π orbital hybridisation with a similar number of Co atoms. A spin polarisation of 0.1 − 1 μ_B_ per hexagon‐pentagon unit has been observed on fullerenes grown on ferromagnetic metal substrates.^[^ ^12^ ^]^

The structural and electronic properties of H_2_Pc molecules and their interaction with the Co surface were evaluated using density functional theory (DFT) calculations. In the optimized structure, the Co atoms beneath the Pc molecule were found to show slight displacements (Figure S2, Supporting Information), indicating a perturbation due to chemisorption. The PDOS (projected density of states) peaks of Co d‐orbitals and C‐p orbitals were found to overlap substantially in the same energy range (Figure S3, Supporting Information), indicating hybridization between the metal and the molecule at the hexagonal‐pentagonal unit. The strong in‐plane anisotropy in Pt/Co/molecular samples at 10 K is clear from changes in the remanence and coercivity in measured hysteresis loops at different angles of magnetic field with respect to the sample plane (Figures S4, Supporting Information). The radical modifications in the magnetic properties of the Co layer are attributed to changes to the valence state of Co close to the surface due to charge transfer and metal‐molecule orbital hybridisation (see Table S1, Supporting Information).

Further to the low‐temperature molecular hybridisation induced hardening, a spin reorientation transition at higher temperatures is evidenced by the in‐plane remanence curves shown in Figure 1e. The onset temperature in 1.7 nm thick Co films ranges from (200 ± 5) K to (285 ± 5) K depending on the molecular overlayer. Although the central‐ions are not part of the hexagon‐pentagon units that hybridise with the metal to lead to observed effects, we believe they may play a role in influencing the degree of hybridisation of the π‐orbitals in the hexagonal‐pentagonal units, hence leading to the observed ranges of temperatures for planar magnetisation onset. A perpendicular easy axis at *T* = 400 K is confirmed in OOP hysteresis loops for all samples (Figure 1f). Accompanied by the onset of the PMA, there is an enhancement in the OOP coercivity for all organic molecules when compared to the reference sample. This agrees with previous room temperature measurements in C_60_,^[^ ^27^ ^]^ although the magnitude of the effect is molecule specific.

### 2.2 Cobalt Thickness and Electrical Current Induced Tunability of Spin‐Reorientation Transition

**Figure 2:**
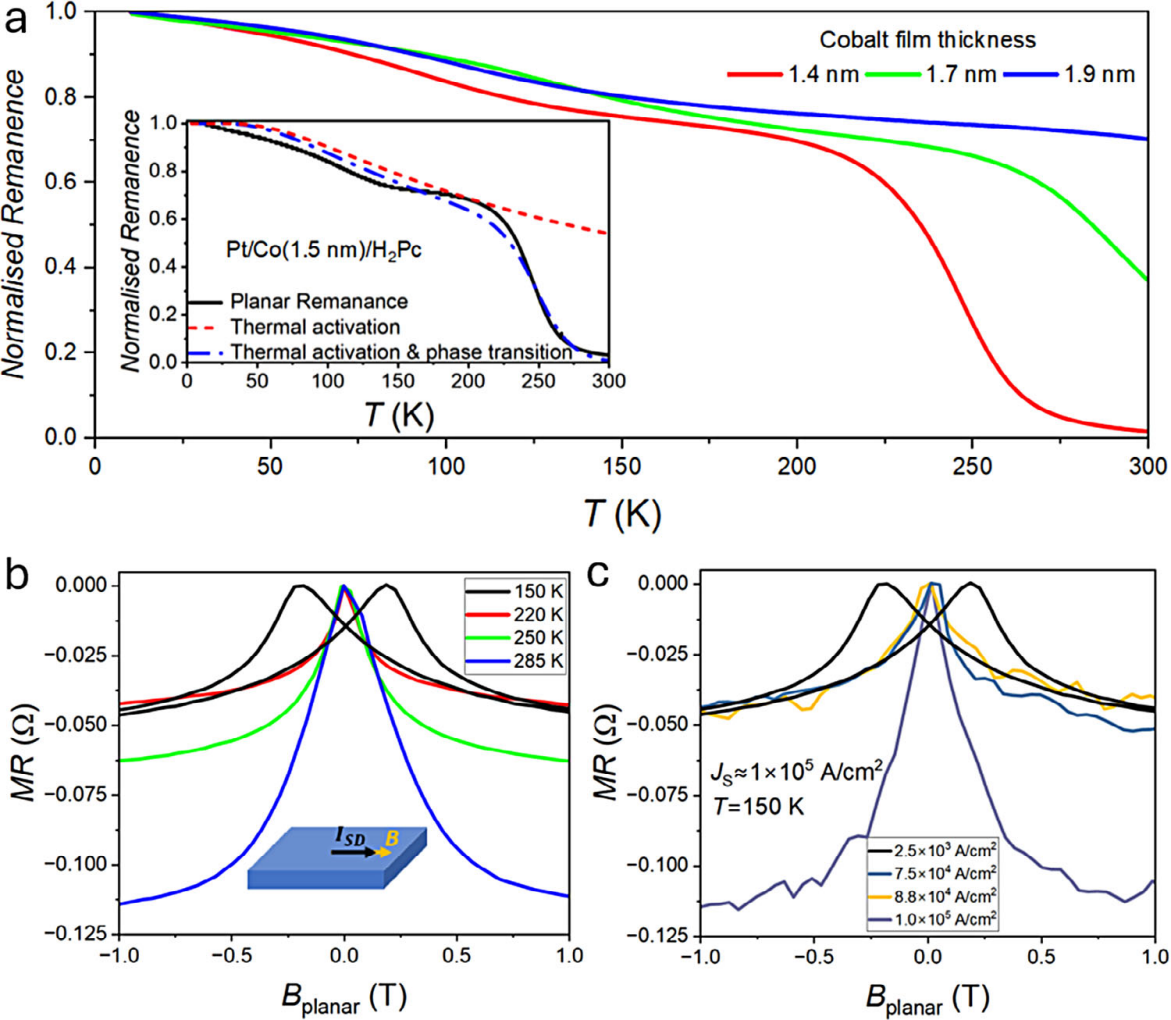
Cobalt thickness dependence of the spin reorientation transition and its electrical current control. a) Planar magnetic remanence curves showing Co thickness dependent spinreorientation transition onset at Pt/Co/CuPc metallo‐molecular structures, with the inset of phenomenological fits to a remanence curve for a Pt/Co/H_2_Pc structure. b) Temperature dependence of magnetoresistance on a Pt/Co(1.7 nm)/CoPc structure measured with a current density of 2.5 × 10^3^ A cm^−2^ during Anisotropic Magnetoresistance (AMR) field sweeps, with the field applied parallel to the electrical current (the inset schematic), showing greater negative magnetoresistance as temperature is increased and spin reorientation transition takes place. c) AMR field sweeps measured at varying current densities on the Pt/Co(1.7 nm)/CoPc structure at T = 150 K, suggesting an induced spin reorientation transition at a switching current density of ≈ 1 × 10^5^ **A** **cm** ^−2^.

The tunability of the spin reorientation transition was studied for Co films with thicknesses ranging between 1.4 and 1.9 nm and interfaced with CuPc. We find that the switching temperature increases with increased Co film thickness, **Figure** **2**a. For Co films of less than 1.4 nm thickness, OOP magnetisation was exhibited at all measurable temperatures up to 400 K. This indicates that the spin–orbit mediated magnetic coupling of the bottom Pt interface is stronger but has a faster thickness decay in comparison with the top molecular interface effect.

The in‐plane easy axis onset can be approximated by a combination of thermally activated remanence with a logistic function (Note S1, Supporting Information). The fit yields an activation energy on the order of 18 meV for a Pt/Co(1.5 nm)/H_2_Pc(15 nm) interface (the inset in Figure 2a). Switching temperatures, defined in the phenomenological model as the point at which the IP magnetisation is 50%, measured in different samples, were found to be as low as (245 ± 5) K, with an upper limit above 300 K (Figures 1e and 2a; Table S2, Supporting Information). The Pt(111)/Co interface anisotropy constant in the absence of molecules was estimated to be ≅0.8 mJ m^−2^ below *T*_S_ (Figure S6, Supporting Information). This energy needed to switch the anisotropy axis is promising for low‐power operation in scalable devices. The thermally activated remanence at *T*<*T*_S_ suggests that the magnetisation axis is settled, with a competition between the temperature‐dependent in‐plane interfacial molecular anisotropy mediated by hexagon‐pentagon π‐3d hybridisation, and the 5p‐3d hybridised Pt/Co interface. The spin–orbit coupling induced perpendicular anisotropy generated at the bottom surface dominates at *T*>*T*_S_ due to molecular vibrations, rotation, and repositioning, reducing the metallo‐molecular coupling.

The narrow temperature window across which the transition occurs, and its tunability ≈300 K depending on the Co‐layer thickness, points us toward electrical control of the spin reorientation. To test this, we carried out temperature dependent Anisotropic Magneto‐Resistance (AMR) measurements near *T*_S_ in a Pt/Co(1.7 nm)/CoPc sample (Figure 2b). Such AMR measurements, where an in‐plane magnetic field is rotated from parallel (0°) to perpendicular to the current (90°) configuration (Figure S7, Supporting Information), have been utilised to monitor spin‐reorientation transitions in smaller samples of exfoliated systems.^[^ ^28^ ^]^ Below *T*_S_, the in‐plane coercivity at 0° is observed in the butterfly‐like AMR curve. Above *T*_S_, there is an increase in the negative AMR as the magnetisation easy axis switches to out of plane. This enables the AMR measurements to act as a probe of the easy axis direction (Figure S8b, Supporting Information, shows a comparison of AMR vs IP magnetisation remanence). A series of high current magnetic field sweeps were carried out at 150 K, well below the *T*_S_ of 245 K for this heterostructure as shown in Figure 2c. The easy axis switching from planar to OOP direction was observed at a current density of *J_S_* =  1 × 10^5^ A cm^−2^. We note the abrupt jump in negative magnetoresistance from −0.046 to −0.114 Ω within a current window of ± 1.2 × 10^4^ A cm^−2^ (see Figure S9, Supporting Information magnetoresistance transition at larger current windows). The origin of the current induced easy axis switching is Joule heating of the sample above *T*_S_ (Figure S8c,d, Supporting Information). The main technological relevance for Joule heating induced switching would be in heat‐assisted technologies such as Heat Assisted Magnetic Recording (HAMR), and comparison can be made with electrically controlled spin reorientation transitions demonstrated in magnetic semiconductors.^[^ ^8^ ^]^ We note that the obtained switching current density is an order of magnitude lower for what has been calculated for achieving easy axis switching in proposed spin reorientation assisted spin transfer torque devices^[^ ^29^ ^]^ and, for example, the current induced antiferromagnetic to ferromagnetic transition in FeRh devices.^[^ ^30^ ^]^

### 2.3 X‐Ray Spectroscopy Probing of the Spin‐Reorientation Transition

**Figure 3:**
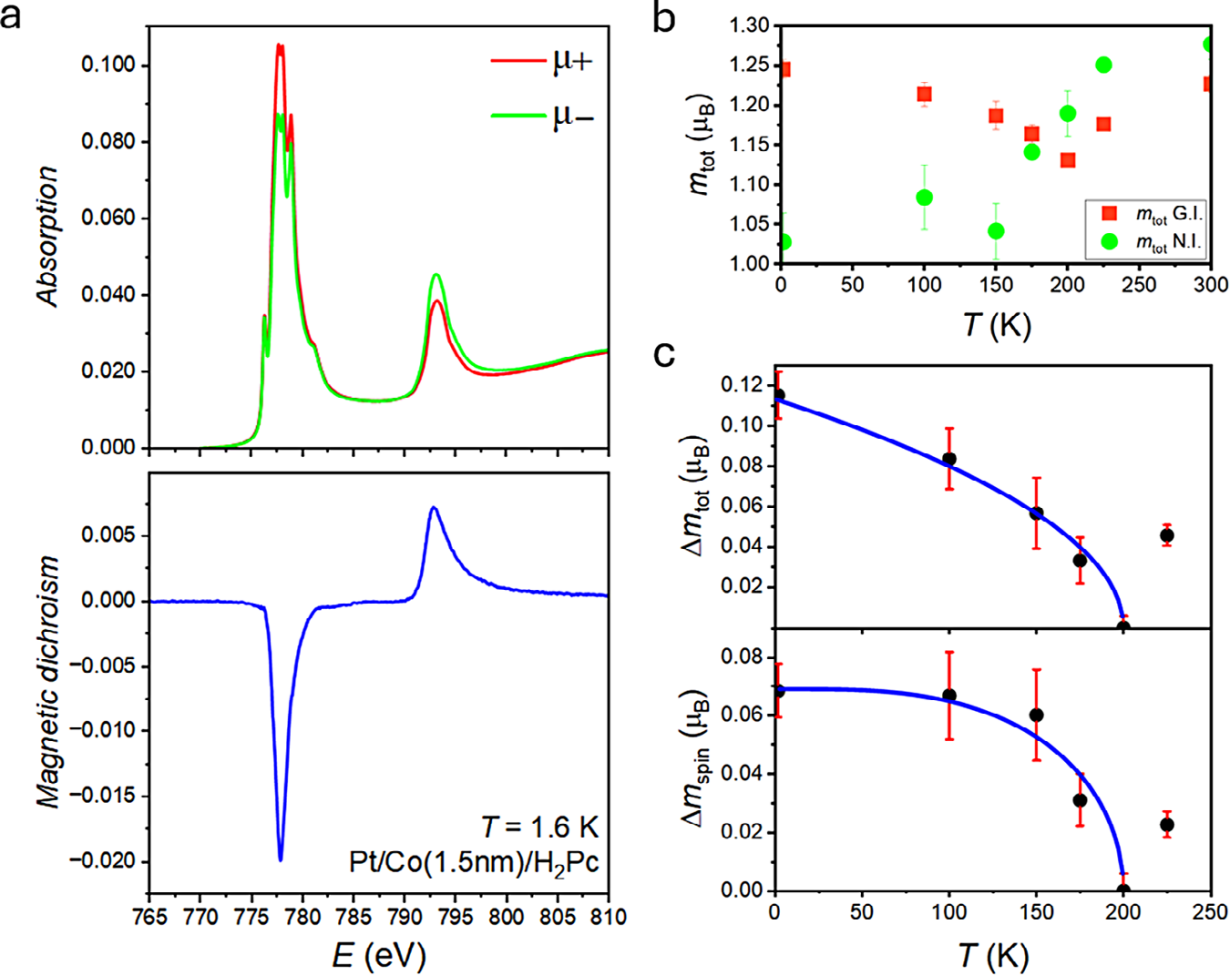
XAS and XMCD measurements in a finite saturating field on a Pt/Co(1.5 nm)/H_2_Pc structure. a) Circular polarisation dependent X‐ray absorption spectrum and the corresponding magnetic dichroism measured at Co edge at 2 T in grazing incidence, showing Co to be in 2+ state due to molecule chemisorption. b) Extracted total magnetic moment in grazing (GI) and normal incidences (NI), where a planar magnetisation onset below T = 200 K is evidenced as GI total moment overtakes NI. c) Change in total moment and spin moment in planar orientation fitted with scaling fits of (*T* − *T_C_*)^1/2^ and $tanh(1.74\sqrt{\left(T_{c}/T^{-1}\right)})$, respectively, with *T_C_* being 200 K.

In order to unravel the physics of the phase transition, X‐ray Absorption Spectroscopy (XAS) and X‐ray Magnetic Circular Dichroism (XMCD) measurements were carried out at the Co L_2,3_ edges in Pt/Co(1.5 nm)/H_2_Pc heterostructures. The XAS spectra indicate that some of the Co atoms have a 2+ valence, as shown in **Figure** **3**a, and this is the case for at all temperatures up to 300 K (see Figure S12, Supporting Information for room temperature XAS/XMCD spectra). The charged state was absent in reference films without molecules (Figure S13, Supporting Information). To rule out an effect due to oxidation, magnetic dichroism measurements were carried out on an oxidised sample (Figure S14, Supporting Information). A comparison of dichroism signals of pristine and oxidised Co/H_2_Pc interfaces at *T* = 2 K is shown in Figure S15 (Supporting Information). Our model estimates 15% Co^2+^ at the surface of the molecule hybridised pristine sample (with 0 eV 10Dq value). We find that in the case of the oxidised sample, both the XAS and the XMCD spectra are instead described by an entire Co^2+^ layer, modeled with a 10Dq value of 0.7 eV (see Figure S16, Supporting Information). The positively charged state and reduced moment are supported by DFT calculations (see Table S1, Supporting Information) with the electron depletion persisting several Co layers below the organic molecule. The strong π‐d orbital hybridisation and chemisorption^[^ ^11^ ^]^ may be enabled by the ultrahigh vacuum (10^−10^ mbar) during the molecule sublimation on the metal surface.

The spectra were analyzed using sum rules^[^ ^31^ ^]^ as shown in Figure 3b,c. (see Figure S17, Supporting Information for sample integrated XAS/XMCD spectra curves). Further to magnetometry, the easy axis switching can also be observed in the total magnetic moment of Co ions, *m_tot_*, derived from XMCD measurements in normal (OOP) and grazing (planar) incidence (Figure 3b). The in‐plane *m_tot_* overtakes the perpendicular *m_tot_* below 200 K. Evidence of such magnetisation axis switching in extracted *m_tot_* is absent on degraded films which is a further finding against oxidation. (see Figure S20, Supporting Information). Figure 3c shows the change in planar *m_tot_* and spin moment, *m_spin_* from the parameters transition onset point at 200 K. The (*T* − *T_C_*)^1/2^ scaling relation predicted for frustrated Kondo spin lattice systems^[^ ^32^, ^33^ ^]^ is found to describe Δ*m_tot_* as a function of temperature. The change in *m_spin_* was found to be well described by a weak coupling many‐body fit, $tanh(1.74\sqrt{\left(T_{c}/T^{-1}\right)})$. The physical mechanism behind the phase transition is likely correlated with long‐range spin interactions between the hexagon‐pentagon hybridisation sites,^[^ ^12^ ^]^ as previously discussed in STM studies of metallo‐molecular interfaces.^[^ ^17^, ^19^, ^34^ ^]^

### 2.4 THz Time‐Resolved Magneto‐Optical Kerr Effect (MOKE) Spectroscopy of the Spin‐Reorientation Transition

**Figure 4:**
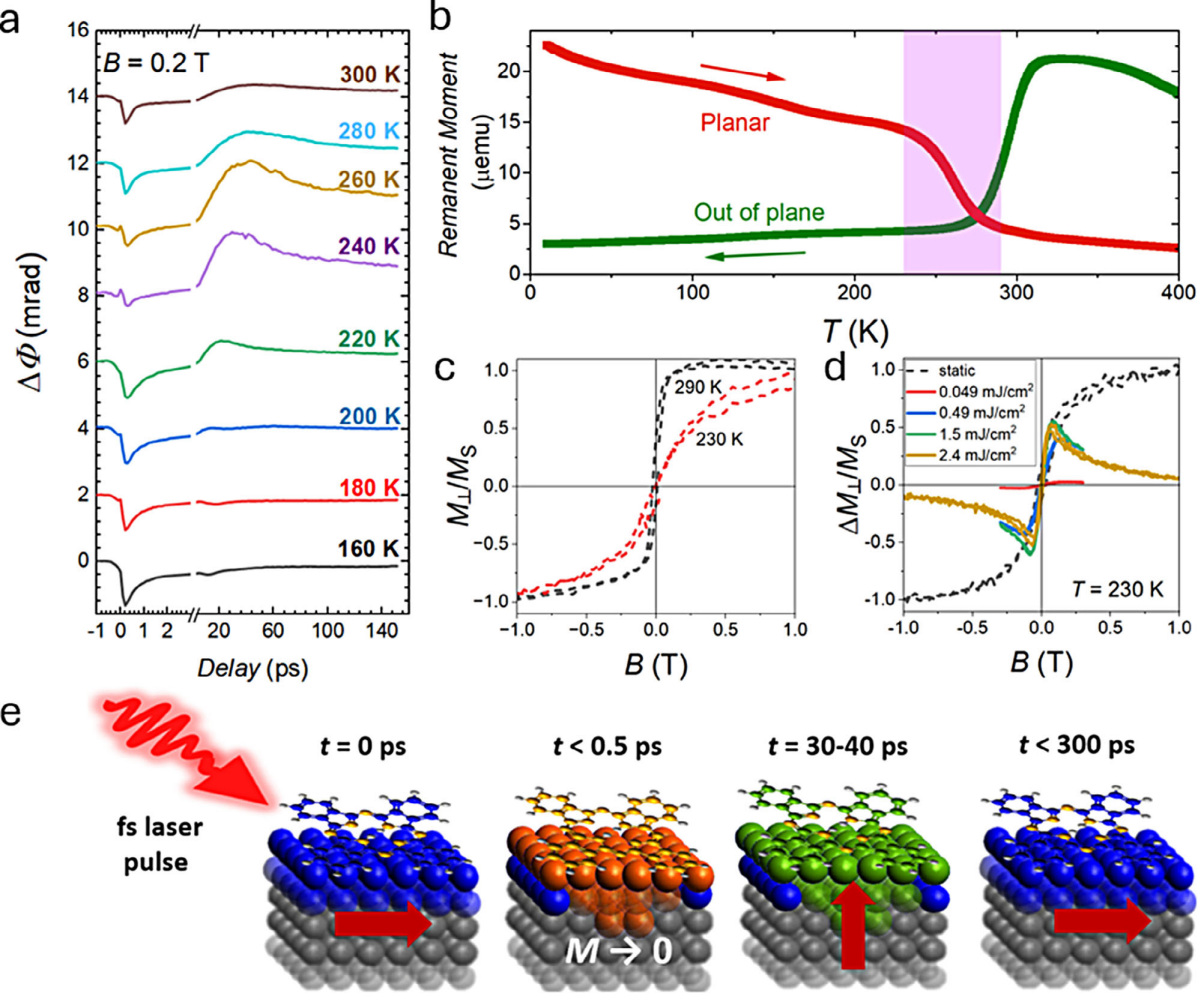
THz magneto‐optical Kerr effect (MOKE) spectroscopy of the spin reorientation transition in Pt/Co(1.4 nm)/H_2_Pc structure. a) Temperature dependent polar time‐resolved Kerr angle transients corresponding to the out‐of‐plane magnetisation measured at an excitation fluence of 0.12 mJ cm^−2^, showing an onset of optical‐pulse‐induced out‐of‐plane spin‐reorientation transition between 220 and 280 K. b) Planar and perpendicular magnetic remanence curves measured on the metallo‐molecular structure with the spin reorientation transition temperature window (from 230 to 290 K) highlighted in pink. c) The out‐of‐plane static polar MOKE hysteresis around the transition window at 230 and 290 K, showing the easy axis emergence on the latter. d) The normalised field‐dependent magnitude of the transient out‐of‐plane magnetisation, Δ*M*_⊥_, in comparison to the static out‐of‐plane magnetisation, Δ*M*_⊥_, at the optimal *T*  = 230 K as a function of the excitation fluence specified in the legend. e) Schematic illustrating the picosecond spin reorientation transition dynamics (suggested in a) with the planar magnet demagnetisation below 0.5 ps followed by the spin reorientation transition after the laser‐induced heating on a 30–40 ps timescale, and planar magnetisation re‐emergence below 300 ps.

The sharp nature of the spin reorientation transition suggests possible applications in ultrafast devices, such as heat assisted memory systems, where switching times below 1 ns are needed.^[^ ^7^, ^35^ ^]^ We measured the timescales involved in the easy axis switching using time‐resolved magneto‐optical Kerr effect spectroscopy (TR‐MOKE). The magneto‐optical Kerr effect transients measured upon excitation with a 50 fs laser pulse (see Experimental Section for more detail and Figure S21, Supporting Information for the setup schematic) with the magnetic field and laser beam nearly perpendicular to the heterostructure plane are shown in **Figure** **4**a. As depicted in Figure 4e, the initial demagnetisation of the sample is observed on a sub‐picosecond timescale, corresponding to the negative part of the TR‐MOKE transient. Near the spin reorientation transition, 230–290 K as shown in Figure 4b,c, the positive part of the TR‐MOKE transient indicates an increase of the OOP magnetisation with a rise time of a few tens of picoseconds (≈40 ps at *T* = 260 K with a weak excitation fluence of 0.12 mJ cm^−2^). The onset OOP magnetisation was found to decay exponentially with a sub‐300 ps decay constant (see Figure S22, Supporting Information, for field dependence at a given temperature). Further data on the dependence of the light‐induced transient OOP magnetisation on magnetic field and excitation fluence is shown in Figure 4d. The data indicate that at a moderate excitation fluence of 0.5 mJ cm^−2^ OOP magnetisation onset of ≈ 40% can be achieved (with respect to the static saturated value), exceeding 50% at 1.5 mJ cm^−2^. The rise time of the OOP magnetisation, however, which increases with increasing excitation fluence (see Figure S23, Supporting Information) is ≈ 100 ps at 0.5 mJ cm^−2^. Our data suggests a parameter of space for novel heat assisted technologies, where optimisation could be carried out to achieve optically induced spin reorientation transition at remarkably low fluences ranging down to 0.12 mJ cm^−2^. This is an order of magnitude smaller than those first used in HAMR systems, for example, to write/erase data,^[^ ^36^ ^]^ second all optical helicity independent switching systems to switch magnetisation direction,^[^ ^37^, ^38^, ^39^, ^40^, ^41^ ^]^ and lastly magnetic tunnel junction structures demonstrating spin reorientation transitions.^[^ ^42^ ^]^

## 3 Conclusion

In conclusion, a low‐energy, ultrafast unconventional spin reorientation transition has been observed near room temperature in Pt/Co/molecular heterostructures with competing magnetic interactions. The transition onset and window are controlled by the ferromagnet thickness and the molecule used. For an appropriate ferromagnetic film thickness range, the molecular interface coupling induced in‐plane magnetisation dominates at low temperatures, whereas the perpendicular magnetisation arising from spin–orbit coupling at the Pt interface is dominant at higher temperatures. The magnetisation easy axis direction is therefore not fixed by the structure composition but can be rotated from in‐plane to out‐of‐plane with ultralow power, paving the way to low‐power information storage and memory applications. The electric switching of the magnetisation easy axis is demonstrated to be possible via local heating with a current density that is an order of magnitude lower (≈ 1 × 10^5^ A cm^−2^) than those used to, e.g., introduce antiferromagnetic – to ferromagnetic transition in FeRh,^[^ ^30^ ^]^ or the densities calculated for spin‐reorientation assisted spin‐transfer torque system proposals.^[^ ^29^ ^]^ In heat assisted technologies, optical or other local heating mechanisms can be used to switch such structures with a local temperature change of just 50 K. Compared to the existing HAMR systems, where approaching toward Curie temperatures is necessary, order of magnitude lower laser fluences of ≈0.12 −0.5 mJ cm^−2^ are shown to be enough for optically induced spin reorientation transition as well as an operation timescale of sub 300 ps. Demonstrated lower laser fluence is also found to be an order of magnitude lower than systems showing all optical helicity independent switching. In future work, further optimisation of energy and timescales should be possible by tuning substrate and film thicknesses, as well as studying other magnetic and molecular materials. By tuning the Co thickness to higher values, spin reorientation transition temperature can be tuned above room temperature, creating a platform for practical memory devices. XAS/XMCD data measured at Co edge evidence the onset of anisotropy switching at the atomic level, where the in‐plane magnetisation can be attributed to correlation of spin moments at the molecular interface and be described by a scaling fit. Beyond the single ion Kondo effect measured in polycrystalline gold molecule interface films,^[^ ^43^ ^]^ the candidate physical mechanism behind the observed planar magnetic phase transition is a temperature dependent onset of a correlated spin lattice effect, arising due to a hybridised carbon supramolecular lattice of spins formed at the metallo‐molecular interface in ultrahigh vacuum.^[^ ^17^, ^19^, ^34^, ^44^ ^]^

## 4 Experimental Section

### Thin Film Growth and Characterization

Thin‐film structures were grown on 0.65 mm thick c‐plane sapphire films. The (111) textured Pt layers of ≈4 nm thickness (Figure S24, Supporting Information, shows the XRD peak) were grown at 500 °C with e‐beam evaporation at a growth rate of ≈0.1Ȧ s^−1^. The substrate was then cooled down to room temperature, and the Co‐layer was grown also with e‐beam evaporation technique at a rate of ≈0.1 Ȧ s^−1^. Within the same chamber, organic molecule layers were sublimed onto the Co surface at a pressure of ≈5 × 10^−10^ mbar with a rate of ≈0.3 Ȧ s^−1^, where the thickness of the organic layer was 20 nm. The cap layer was magnetron sputtered on top of the thin film structure, with the material used for capping being Cu with a thickness of ≈15 nm. Films were then structurally characterised using X‐ray reflectivity and X‐ray diffraction. In order to determine the thicknesses of each layer in the thin film heterostructure, in particular the Co layer where the thickness dependence was studied, a calibrated quartz crystal monitor inside the growth chamber and X‐ray reflectivity were used. The thickness for each layer was set using the quartz crystal and then confirmed through a fitting of the X‐ray reflectivity curve (Figure S25, Supporting Information). Transmission electron microscopy characterization of similar structures can be found elsewhere.^[^ ^45^, ^46^ ^]^ Raman characterization of a phthalocyanine metallo‐molecular structure is shown in Figure S26 (Supporting Information).

### Magnetometry

Magnetisation measurements were carried out using SQUID‐magnetometer (MPMS Q.D.), which offers a resolution over 10^−8^ emu accompanied by temperature control.

### XAS/XMCD

X‐ray absorption and magnetic circular dichroism measurements were carried out at BL‐29 BOREAS of the ALBA synchrotron light source.^[^ ^47^ ^]^ For XAS studies, a thinner Cu 3 nm was deposited on the metallo‐molecular structures to prevent attenuation by the Cu cap. The error bars were obtained on magnetisation values determined post sum rule analysis by taking the range of moment values obtained post subtraction of varying values of background signal. The Co^2+^ XAS and XMCD calculations were carried out by crystal field multiplet (CFM) simulations using CTM4XAS.^[^ ^48^ ^]^ 10Dq *=* 0 eV was chosen, assuming the Cobalt was in spherical symmetry. For calculations, the final Slater integrals, F(*pd*), G(*pd*), and F(*dd*) were considered a standard reduction of 80% to their Hartree‐Fock values. The CFM simulations were done at Co^2+^ electronic configuration and simulated for the C_4_ crystal symmetry. The *M* value was chosen as 6 meV while the *D_t_* and *D_s_* remain to zero. XMCD simulations for Co/H2Pc interface were carried out using an 85% composition of Co and 15% composition of Co^2+^ with a 10Dq value of 0. For modeling oxidised sample, CoO spectrum with the crystal field parameter, 10Dq ≈0.7 eV was found to match to the experimental spectra.

### Electronic Transport

4‐probe resistance measurements were carried out in continuous flow cryostats on devices of narrow cleaved strips of films, with a Keithley 6621 DC and AC current source with a Keithley nanovoltmeter being used for current induced switching measurements.

### Time Resolved Magneto‐Optical Kerr Effect (TR‐MOKE)

Measurements were conducted by means of a two‐color pump‐probe setup based on a high repetition‐rate (250 kHz) 50‐femtosecond Ti:sapphire laser amplifier and a split‐coil superconducting 7 T optical magnet with a variable temperature He exchange gas sample insert (see Figure S21, Supporting Information). A part of the output pulse train was used to derive the pump pulses that were either at the laser fundamental (λ = 800 nm, 1.55 eV) or frequency doubled (λ = 400 nm, 3.1 eV photon energy). The probe pulses with a variable time delay were derived from the remaining fundamental pulse train. The reflected‐probe‐beam transient (and static) polarisation rotation was detected by means of a balanced detection using a Wollaston prism and a pair of silicon PIN photo diodes. The pump and probe beams were modulated at two different frequencies in a couple of kHz range with an optical chopper, and a standard lock‐in detection scheme was used to acquire the photodiodes sum and/or differential signal. The magnetic field and the laser beams were nearly perpendicular to the heterostructure plane in a polar TR‐MOKE configuration.

### Density Functional Theory

All first‐principles calculations were performed using the Vienna Ab Initio Simulation Package (VASP) within the projector‐augmented wave (PAW) formalism. The exchange‐correlation functional was described using the generalized gradient approximation (GGA) as formulated by Perdew, Burke, and Ernzerhof (PBE). To accurately capture on‐site Coulomb interactions, the LDA+U (U = 0, J = 0) method was enabled. The Co substrate was modeled as a 4 × 6 (17.96 × 15.06 Å) 4‐layer slab of the (111) surface of FCC Co with 15 Å vacuum in the c direction. A plane‐wave kinetic energy cutoff of 400 eV was employed in a ferromagnetic spin configuration. The 2D Brillouin zone was sampled with a 2 × 2 k‐point grid centred at the Gamma point. Structural relaxation was carried out with a force tolerance of 0.05 eV Å^−1^. The RMM‐DIIS algorithm was used for electronic minimization to accelerate convergence with total energy and force convergence criteria set to 10^−4 ^eV and −0.05 eV Å^−1^, respectively, ensuring an accurate determination of the electronic structure. To capture the magnetic properties of the system, spin polarization was explicitly included, and the initial magnetic moments were defined for Co atoms. To account for dispersion interactions, the DFT‐D3 van der Waals correction was enabled. Bader analysis was performed on the all‐electron charge density by partitioning it into atomic regions using the grid‐based zero‐flux method. The charge integration was performed using the Henkelman algorithm, ensuring an accurate evaluation of atomic charges.

## Conflict of Interest

The authors declare no conflict of interest.

## Supporting information



Supporting Information

## Data Availability

The data that support the findings of this study are openly available in University of Leeds Research Data Repository at https://doi.org/10.5518/1705, reference number [1705].
