# Supplementary material for: Low‐Energy, Ultrafast Spin Reorientation at Competing Hybrid Interfaces with Tunable Operating Temperature
Source: Adv Mater. 2025 Aug 5;37(42):e19192. doi: 10.1002/adma.202419192 (PMC12548515; doi:10.1002/adma.202419192)
Supplement: Supplementary file 1 — Supporting Information [file ADMA-37-e19192-s001.docx]

**Supporting Information**

**Low-energy, ultrafast spin reorientation at competing hybrid interfaces with tunable operating temperature**

*Servet Ozdemir^*^, Matthew Rogers, Jaka Strohsack, Hari Babu Vasili, Manuel Valvidares, Thahabh Haddadi, Parvathy Harikumar, David O’Regan, Gilberto Teobaldi, Timothy Moorsom, Mannan Ali, Gavin Burnell, Bryan J Hickey, Tomaz Mertelj, Oscar Cespedes^*^*

**Supporting Note 1**

In order to model the spin-reorientation transition, the usual thermal activated magnetic remanence model was combined with a logistic-function

$$\left( 1-exp\left( \frac{-E_{a}}{k_{B}T} \right) \right)\cdot\left( \frac{1}{1+\exp\left( -k\left( T-T_{S} \right) \right)} \right)$$

where one can immediately see that the steepness function *k* defines the transition window, that we can re-write as a spin canting temperature window as

$$\left( 1-exp\left( \frac{-E_{a}}{k_{B}T} \right) \right)\cdot\left( \frac{1}{1+\exp\left( -\frac{\left( T-T_{S} \right)}{T_{w}} \right)} \right)$$

with both quantities $T_{S}$ and $T_{w}$ obtainable through a fit of the model to the experimentally observed remanence curves.

**Supporting Information Tables**


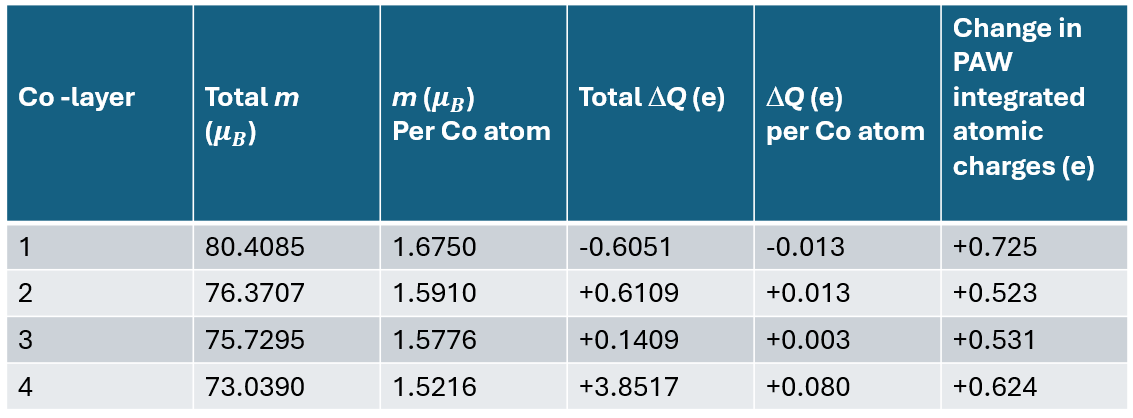


**Table S1**: VASP derived, layer resolved analysis of (PAW-core integrated) magnetic moments (column 2 and 3), change in atomic charges (column 4 and 5) and change in total Bader derived (or PAW integrated) atomic charges (column 6). The sign convention used means a positive value of change in net charge in columns 4-6 means charge depletion. Layer 4 is the topmost H_2_Pc molecule hybridised layer.


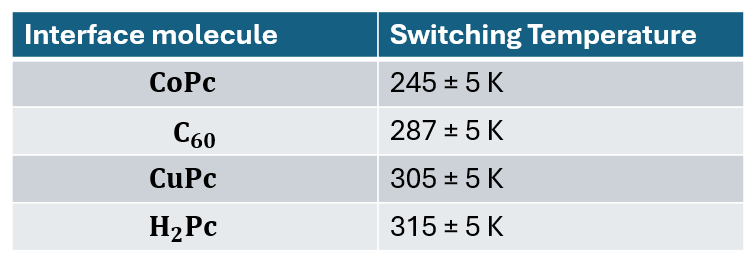


**Table S2**: Extracted switching temperatures on Pt/Co(1.7nm) interfaces using the phenomenological model described in Supporting Note 1.

**Supporting Figures**


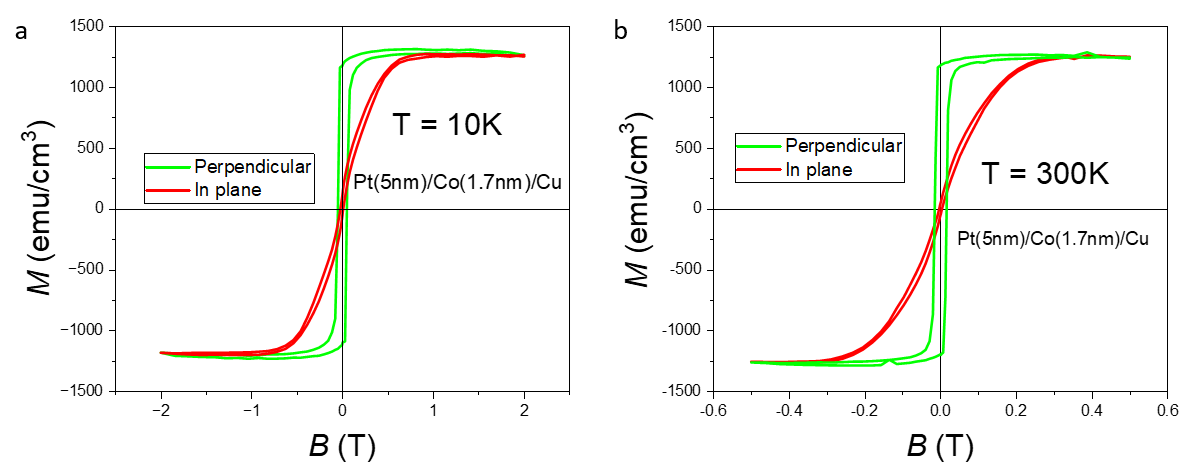


**Figure S1.** Perpendicular magnetic anisotropy reference samples without molecules. Pt/Co(1.7nm)/Cap film measured at perpendicular and planar orientations at **a,** T=10K, and **b,** T= 300K.


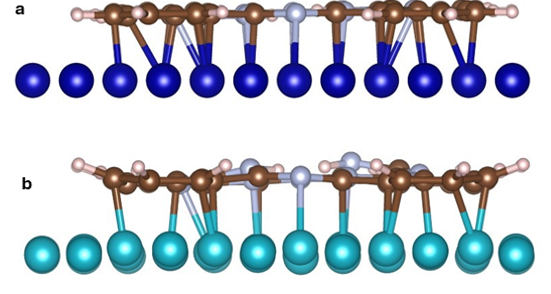


**Figure S2.** Flat lying Pc molecule depicted on Co(111) surface. **a**, initial metal-molecule contact leading to hybridisation **b**, post hybridisation relaxation showing the Co atom distortions.


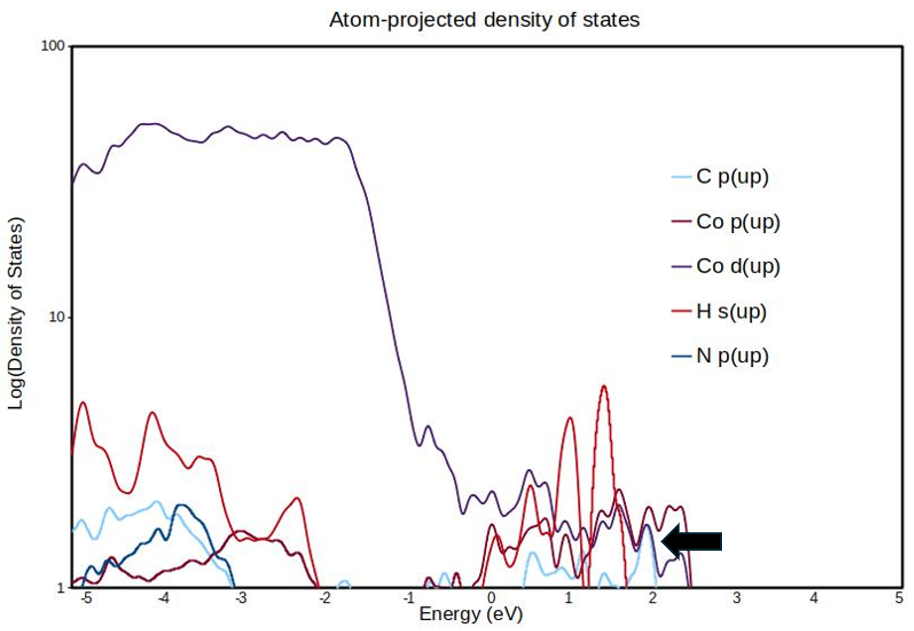


**Figure S3.** Atom projected density of states at the H_2_Pc and Co (111) interface. The region of overlap between Co d and C p orbitals can be seen pointed by the arrow suggesting hybridisation between the respective atoms at the interface.


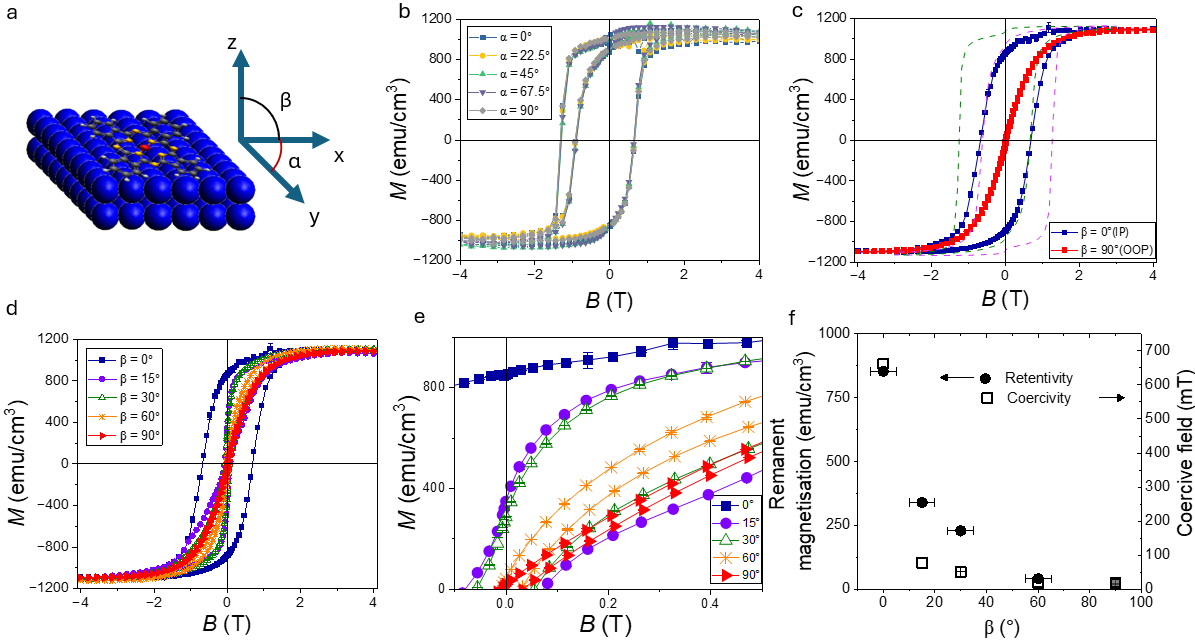


**Figure S4.** Low temperature (T= 10K) magnetic field rotation experiments for magnetic hysteresis on a Pt/Co(1.7nm)/C60 interface. **a,** Thin film schematic and the respective 3 axis labelled x, y and z, with in plane magnetic field rotation angle labelled α, and out of plane magnetic field rotation angle labelled β. **b,** Magnetic hysteresis curves measured at varying in plane rotation angles, α, showing no effect of in-plane rotation on remanent magnetisation and coercive field. **c**, Hysteresis loops measured at different two out of plane rotation angles β = 0° and β=90° suggesting in-plane magnetic anisotropy suggested by higher coercivity and larger remanent magnetisation at zero field for β = 0°(dashed lines correspond to magnetic pinning induced on the sample by field cooling at opposite polarities). **d,** Out of plane magnetic field rotation angle, β, dependence of magnetic hysteresis curves, showing an easy to hard axis switching as the field is rotated from β = 0° to β = 90°. **e**, Magnified version of graph presented on panel d, where smaller remanent magnetisation at zero field is evident at increasing angles β, as field is switched from planar (0°) to perpendicular (90°) orientation. **f**, Remanent magnetisation (retentivity -left y axis) and coercivity (right y axis) as a function of angle β, confirming magnetic anisotropy of the sample to be in-plane when β = 0°.


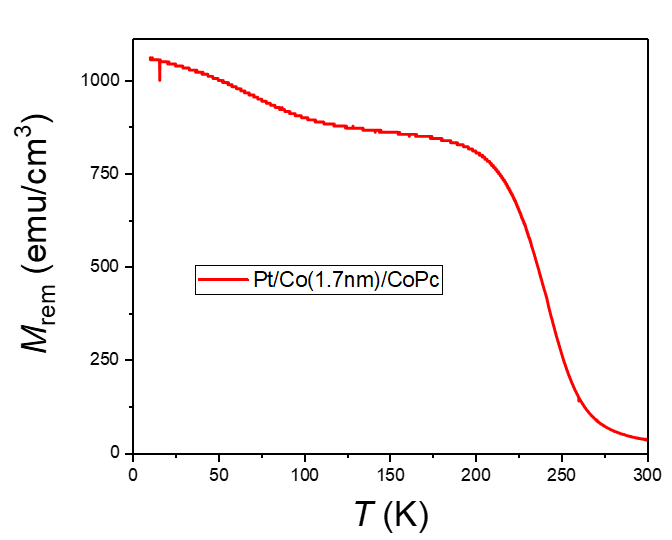


**Figure S5.** Remanent magnetisation measured post field cooling at 2 T, as a function of temperature during warming from 10K to 300K on Pt/Co(1.7nm)/CuPc heterostructure.


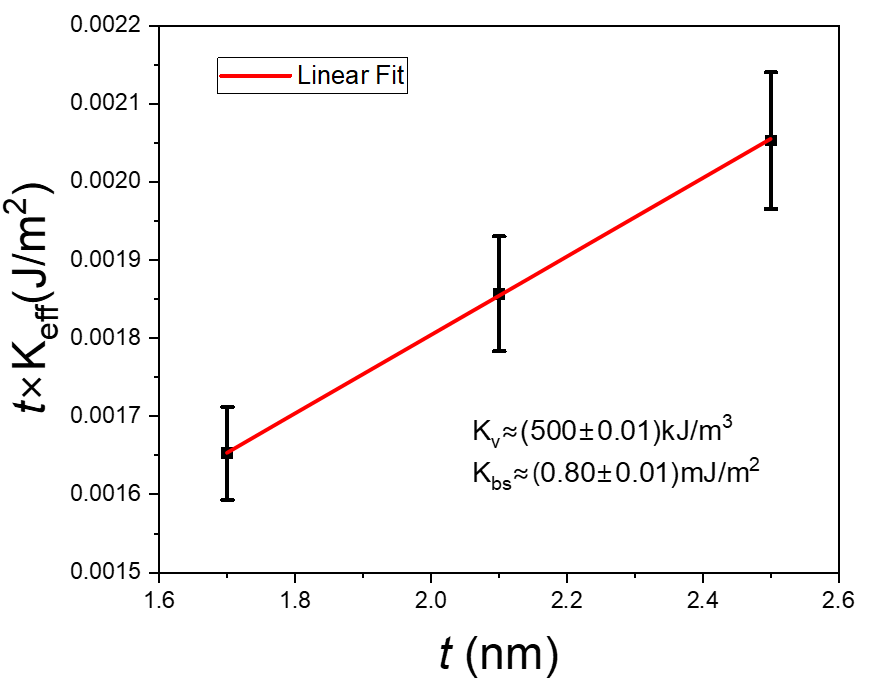


**Figure S6.** Pt/Co interface anisotropy constant estimation. Varying Co thickness hard axis extracted $K_{eff}$, multiplied by $t_{Co}$ plotted as a function of $t_{Co}$ yielding the volume and bottom interface anisotropy energies with slope and intercept respectively**.**


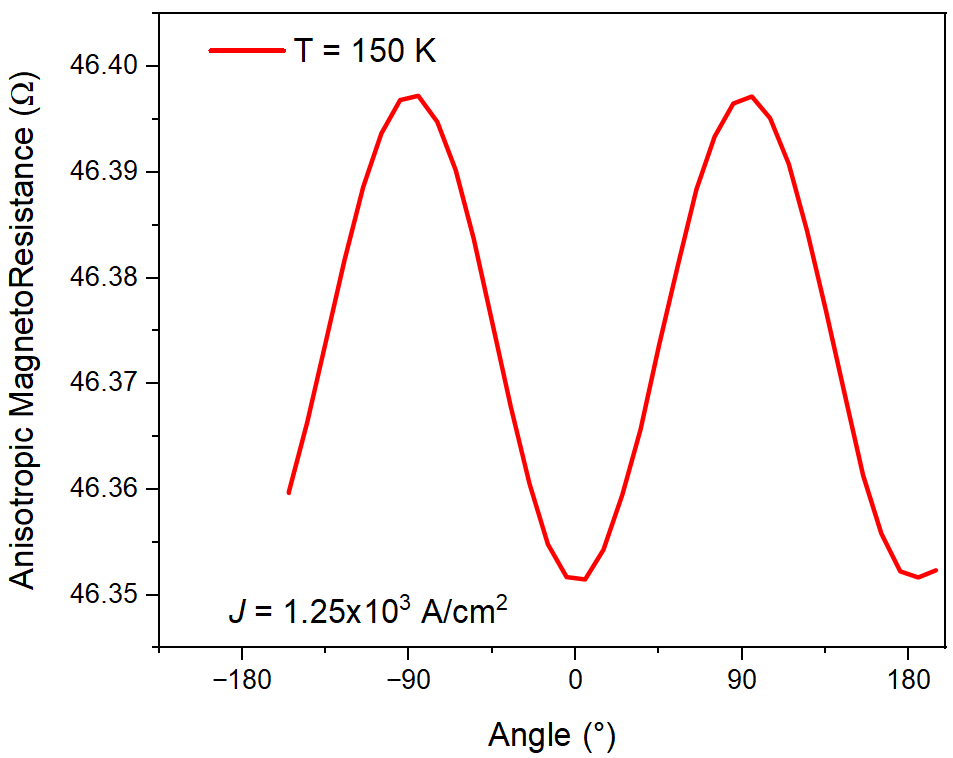


**Figure S7.** Anisotropic magnetoresistance curve measured at 150 K at a 3T field as magnetic field is rotated with respect to current (0 to 180 degrees), with the resistance minima corresponding to angles at which current is aligned to magnetic field.


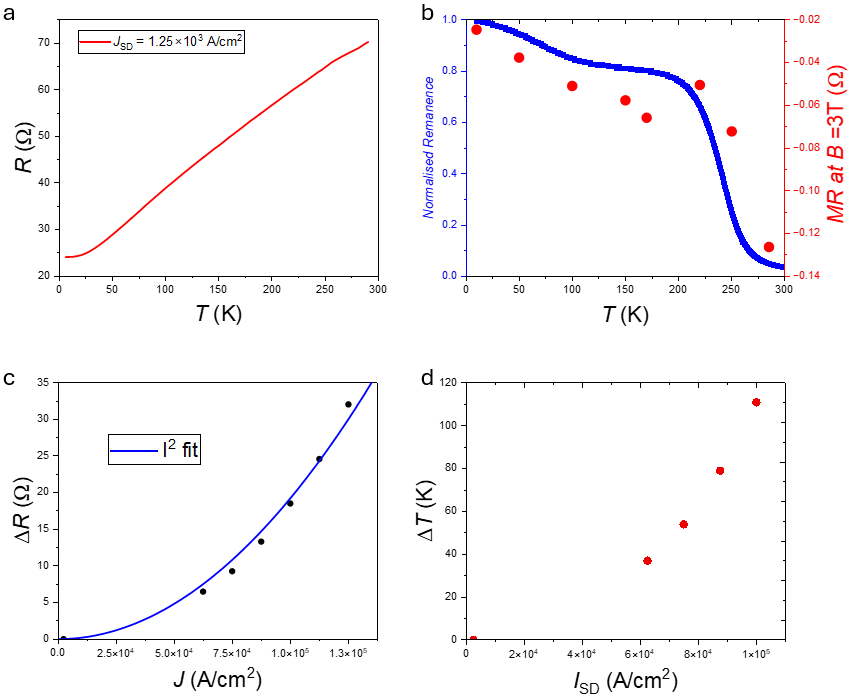


**Figure S8.** Enhanced negative magnetoresistance post spin-reorientation transition and Joule heating of the sample on Pt/Co(1.7 nm)/CoPc heterostructure. **a**, Cooling curve measured on Pt/Co(1.7 nm)/CoPc interface with a current density of $1.25\times{10}^{3} A/\mathrm{cm}^{2}$. **b**, Planar normalised remanence (blue) and magnetoresistance measured at 3 T (red, MR at B = 3 T) planar magnetic field aligned to current (0^o^) as function temperature, showing an enhancement in negative magnetoresistance as easy axis is switched from in plane to out of plane. **c**, Joule heating fit (blue) to the variation in ΔR with ΔR being the change in zero magnetic field resistance due to heating at high current densities, measured at a cryostat temperature of 150 K. **d**, Interpolated temperature change as a function of current density on the Pt/Co(1.7 nm)/CoPc heterostructure, using the cooling curve shown in **a**, and ΔR values shown in **c**.


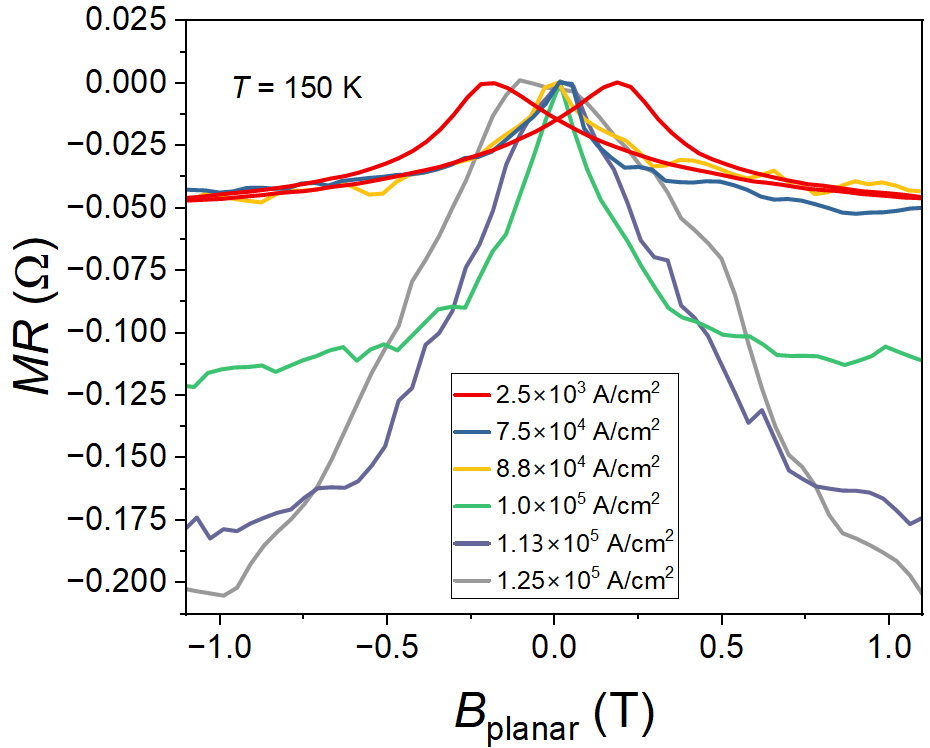


**Figure S9.** AMR magnetic field sweeps carried out at different current densities below switching temperature, showing magnetoresistance corresponding to current densities below a critical switching current ($2.5\times{10}^{3} A/\mathrm{cm}^{2}$ -red, $7.5\times{10}^{4} A/\mathrm{cm}^{2}$ -blue, $8.8\times{10}^{4} A/\mathrm{cm}^{2}$- yellow), and above a critical switching current ($1\times{10}^{5} A/\mathrm{cm}^{2}$-green, $1.13\times{10}^{5} A/\mathrm{cm}^{2}$-purple, $1.25\times{10}^{5} A/\mathrm{cm}^{2}$-grey). The jump in magnetoresistance at 1T is 0.068 Ω, 0.135 Ω, 0.159 Ω at current windows of $\pm1.2\times{10}^{4} A/\mathrm{cm}^{2}$, $\pm2.5\times{10}^{4} A/\mathrm{cm}^{2}$, $\pm3.7\times{10}^{4} A/\mathrm{cm}^{2}$ respectively.


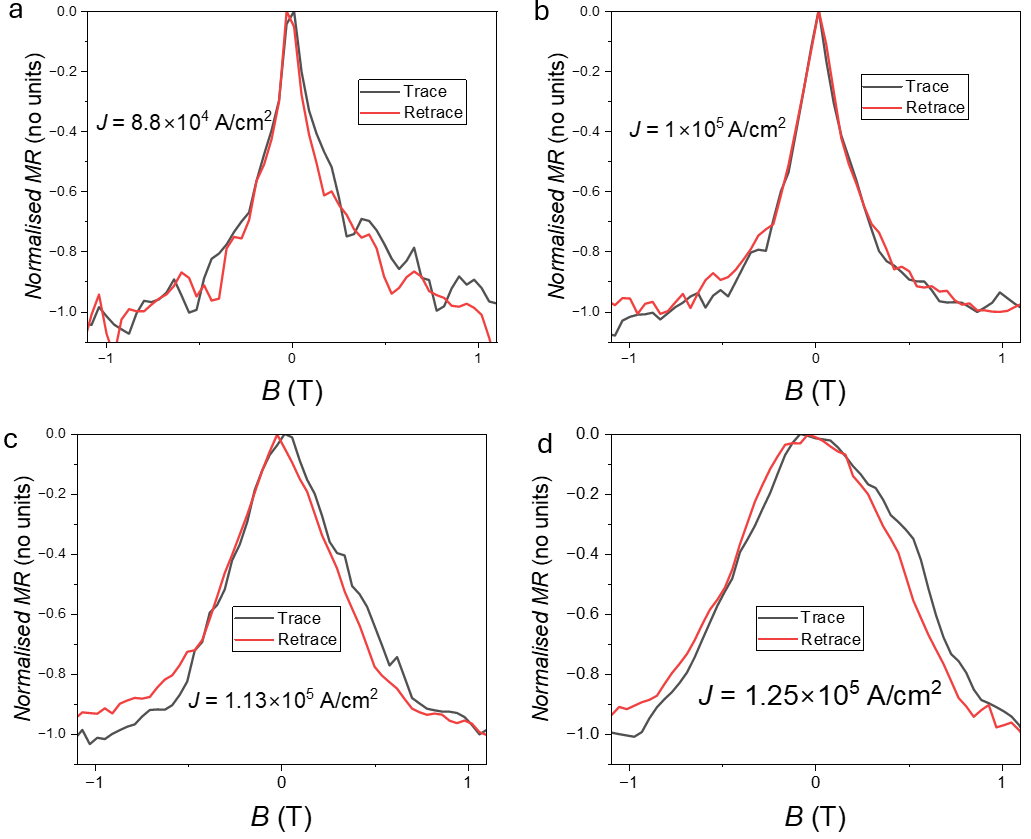


**Figure S10.** Normalised magnetoresistance Trace -Retrace curves measured at T=150 K with various high currents **a**, 8.8 × 10^4^ A/cm² , **b,** 1 × 10^5^ A/cm² **c**, 1.13 × 10^5^ A/cm² and **d**,1.25 × 10^5^ A/cm² showing absence of butterfly-like magnetoresistance, when a Joule heating induced spin-reorientation transition takes place.


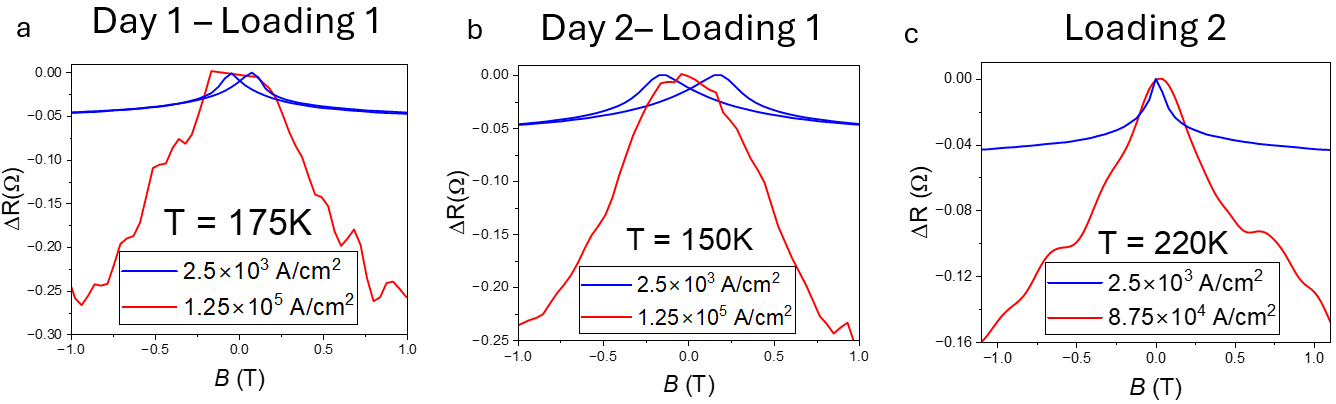


**Figure S11.** AMR magnetic field sweeps carried out at different current densities below switching temperature, showing magnetoresistance corresponding to planar (blue), perpendicular (red) magnetised states, measured at different days in a given cryostat loading **a** and **b**, and measured at a different cryostat loading **c**, suggesting stability of the devices post Joule heating cycles.

**
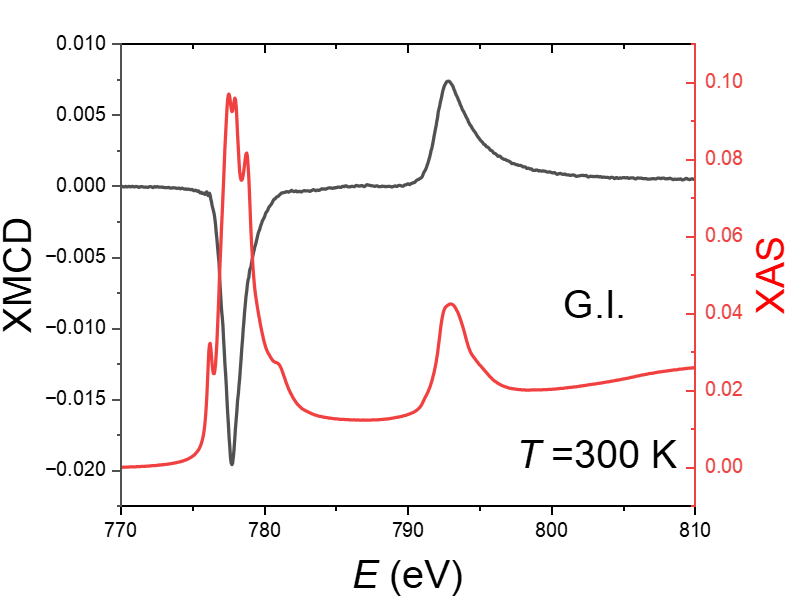
**

**Figure S12.** XMCD (black) and XAS (red) spectrum of Pt/Co(1.5nm)/H_2_Pc sample at room temperature with the XAS spectrum suggesting Co to be in 2+ state.


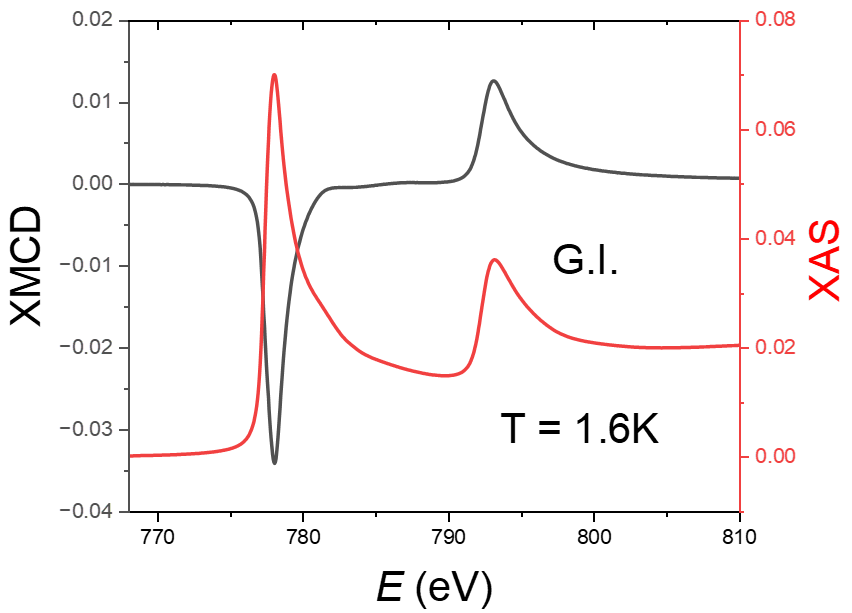


**Figure S13.** XMCD (black) and XAS (red) spectrum at grazing incidence measured on the capped Pt(5nm)/Co(1.5nm) reference sample, with the ionised Co 2+ state shown to be absent.

**
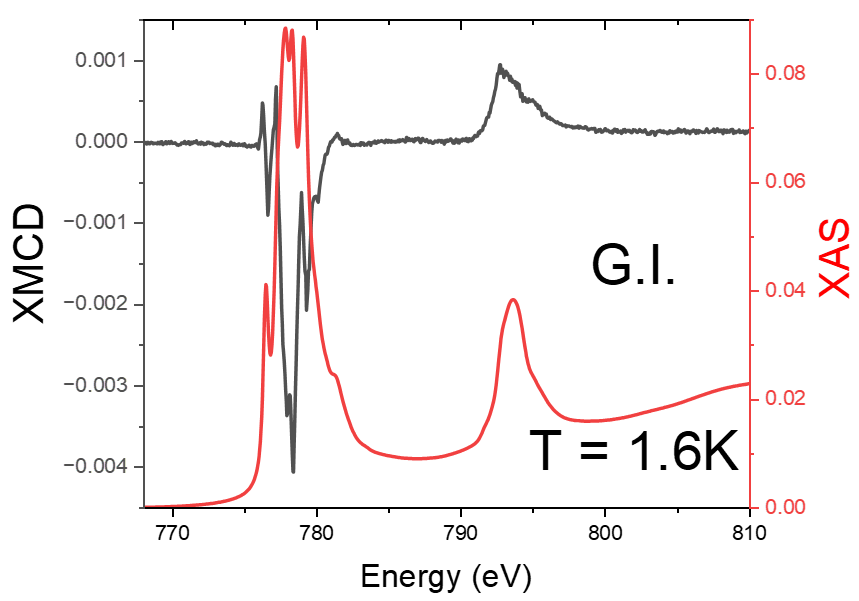
**

**Figure S14.** XAS and XMCD spectrum of improperly capped Pt/Co(1.5nm)/CuPc interface. Disrupted magnetism and an altered Co2+ state due to oxidation is evident.


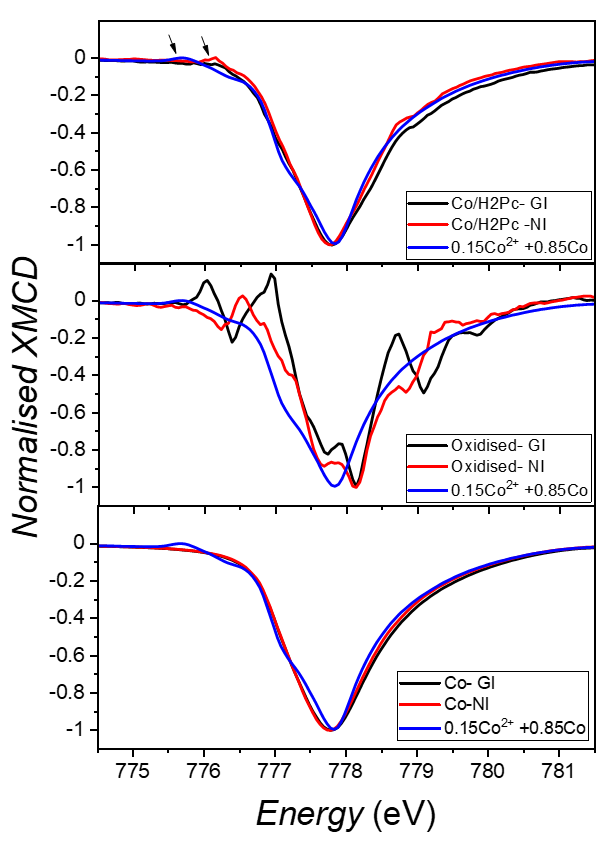


**Figure S15.** Normalised XMCD data (black-G.I., red-N.I.) on Co/H2Pc, oxidised Co, and reference Co on top, middle and bottom graphs respectively plotted with a calculation (blue) that models XMCD spectrum of a 15% Co^2+^ and 85% Co sample, with the 10Dq value used for Co^2+^ being 0 eV.


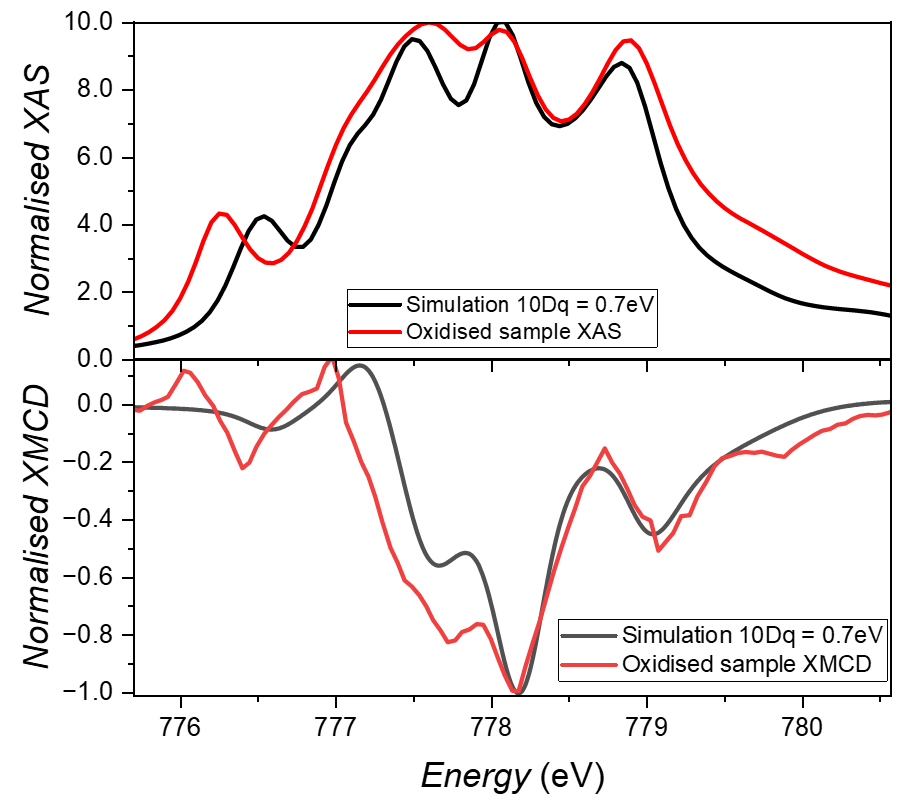


**Figure S16.** Normalised experimentally measured XAS and XMCD (red curves, top and bottom graphs respectively) on an oxidised sample, plotted with XAS and XMCD CoO simulations (black curves, top and bottom graphs respectively) calculated using a 10Dq value of 0.7eV on an entirely Co^2+^ sample.

**
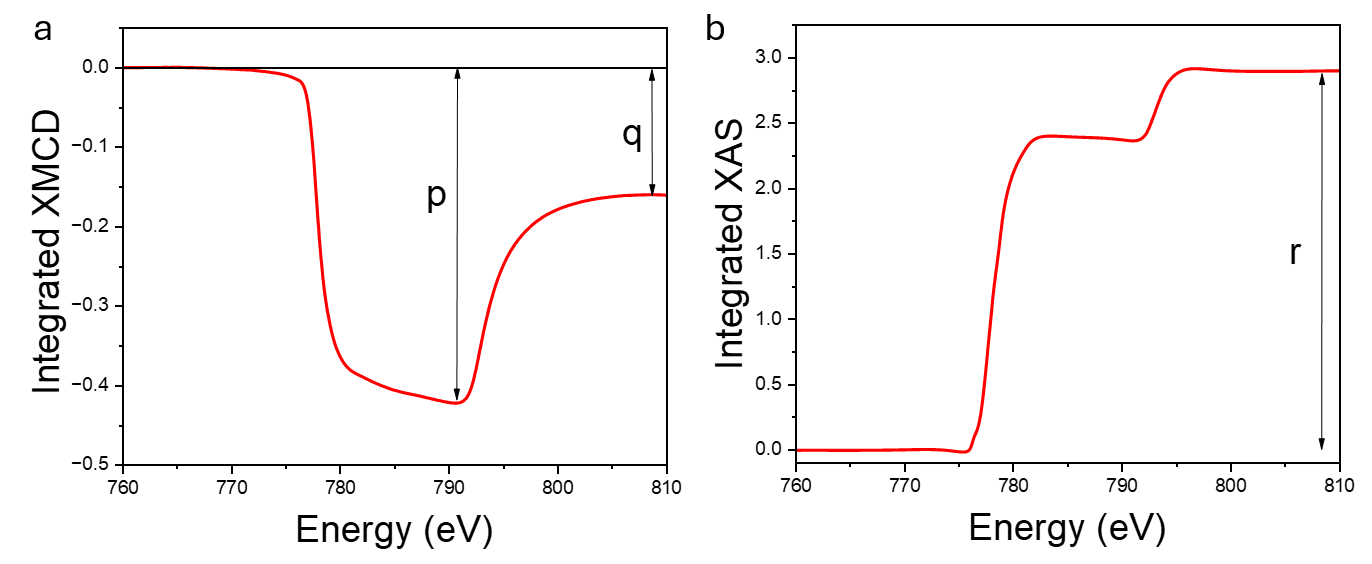
**

**Figure S17.** Integration of **a**, XMCD and **b**, XAS curves measured at grazing incidence at T=1.6K at a magnetic field of 2T on a Pt/Co(1.5nm)/H_2_Pc structure, showing determination of sum rule quantities p, q and r.

**
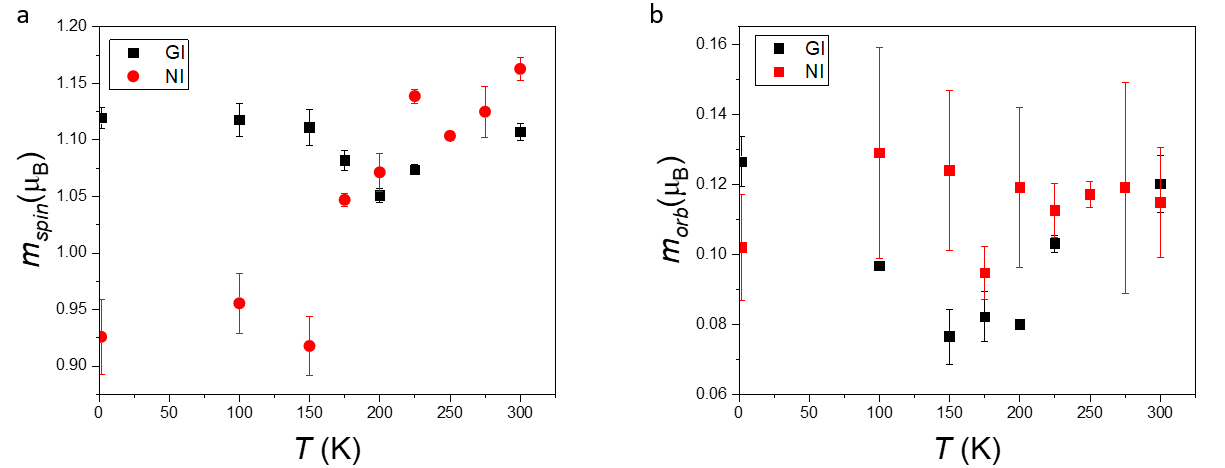
**

**Figure S18.** Further data on Pt/Co(1.5nm)/H2Pc interface reported in the main text. Extracted **a**, spin and **b**, orbital moment components for the Pt/Co(1.5nm)/H2Pc interface.


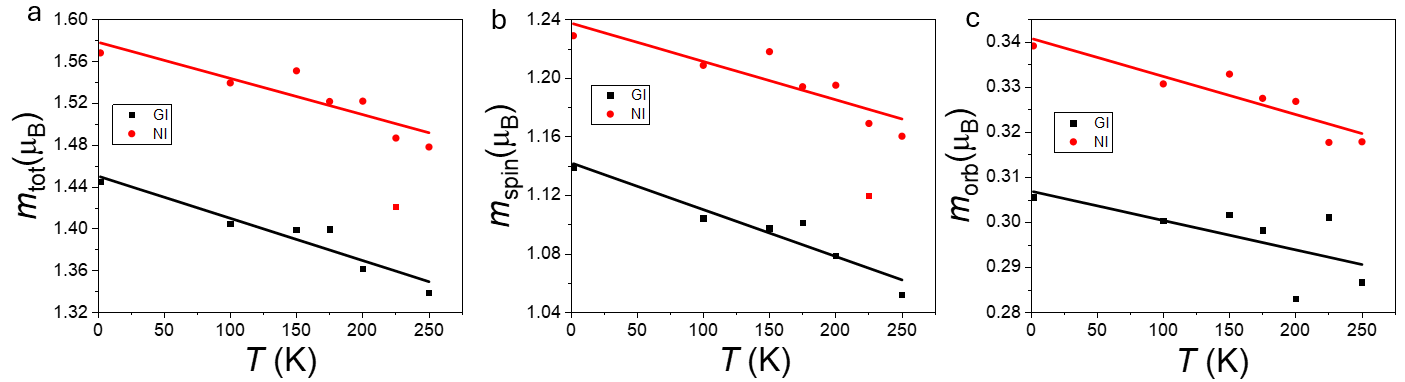


**Figure S19. a**, **b**, **c**, extracted total moment, spin moment and orbital moment on the reference Pt/Co(1.5nm)/Cap sample showing perpendicular magnetic anisotropy at all temperatures.


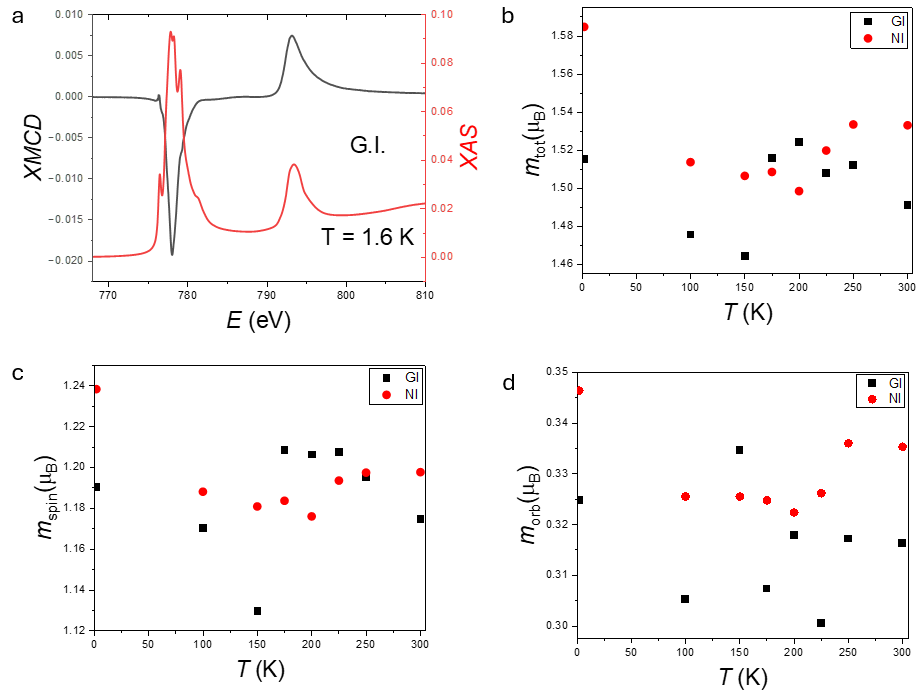


**Figure S20. a,** XAS and XMCD data on a Pt/Co(1.5nm)/CuPc/Cu (3nm) structure measured a year after growth. **b, c, d,** Total magnetic moment, spin moment and orbital moments respectively, with temperature dependence of total magnetic moment showing lack of grazing incidence magnetisation overtaking normal incidence, spin reorientation transition therefore being absent.


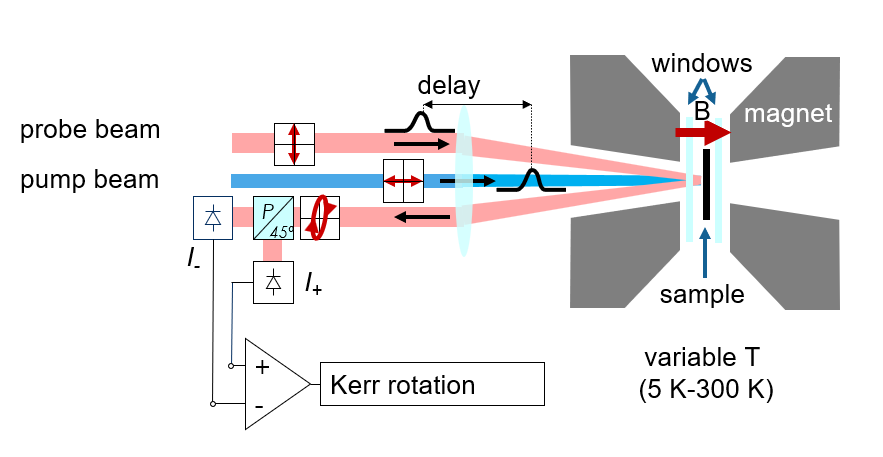


**Figure S21.** Schematic of the time-resolved magneto-optical Kerr effect measurement set up. A linearly-polarised pulsed pump beam, depicted in blue, is focused on a sample by a lens. To probe the OOP sample magnetization a delayed linearly-polarised pulsed probe beam, depicted in light red, is reflected from the sample. The magnetic field is pointing in OOP orientation with respect to sample. The polarisation of the reflected probe beam is then analysed by means of the polarising beam splitter and a pair of photodiode detectors. The four-quadrant squares with the red lines indicate the polarization of the beams at different positions along the path.

**
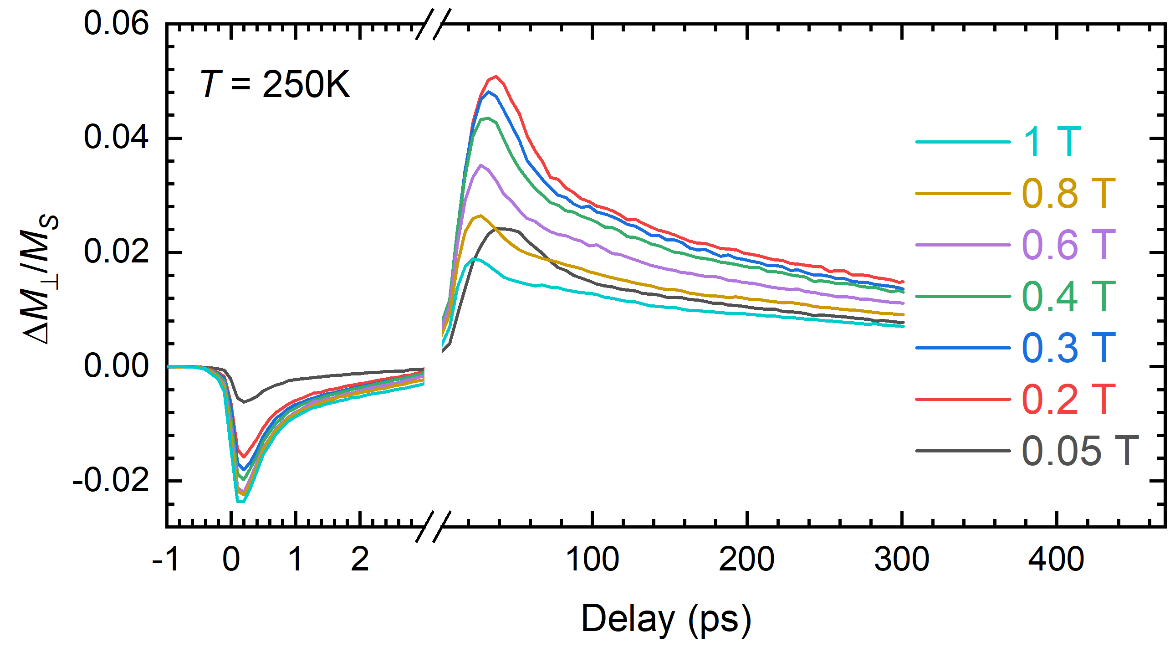
**

**Figure S22.** Magnetic-field dependent time-resolved dynamics at a pump fluence of 0.12 mJ/m^2^ where the magnetic field direction is pointing OOP with respect to sample as depicted in Figure S21. The demagnetisation (0-3 ps) is followed by an increase of the OOP magnetisation, observed as the positive part of the magneto-optical Kerr angle transients (20-300 ps) measured in different external fields at 250 K in a Pt/Co(1.4 nm)/H_2_Pc system. The risetime is decreasing with increasing OOP magnetic field indicating precession dynamics.


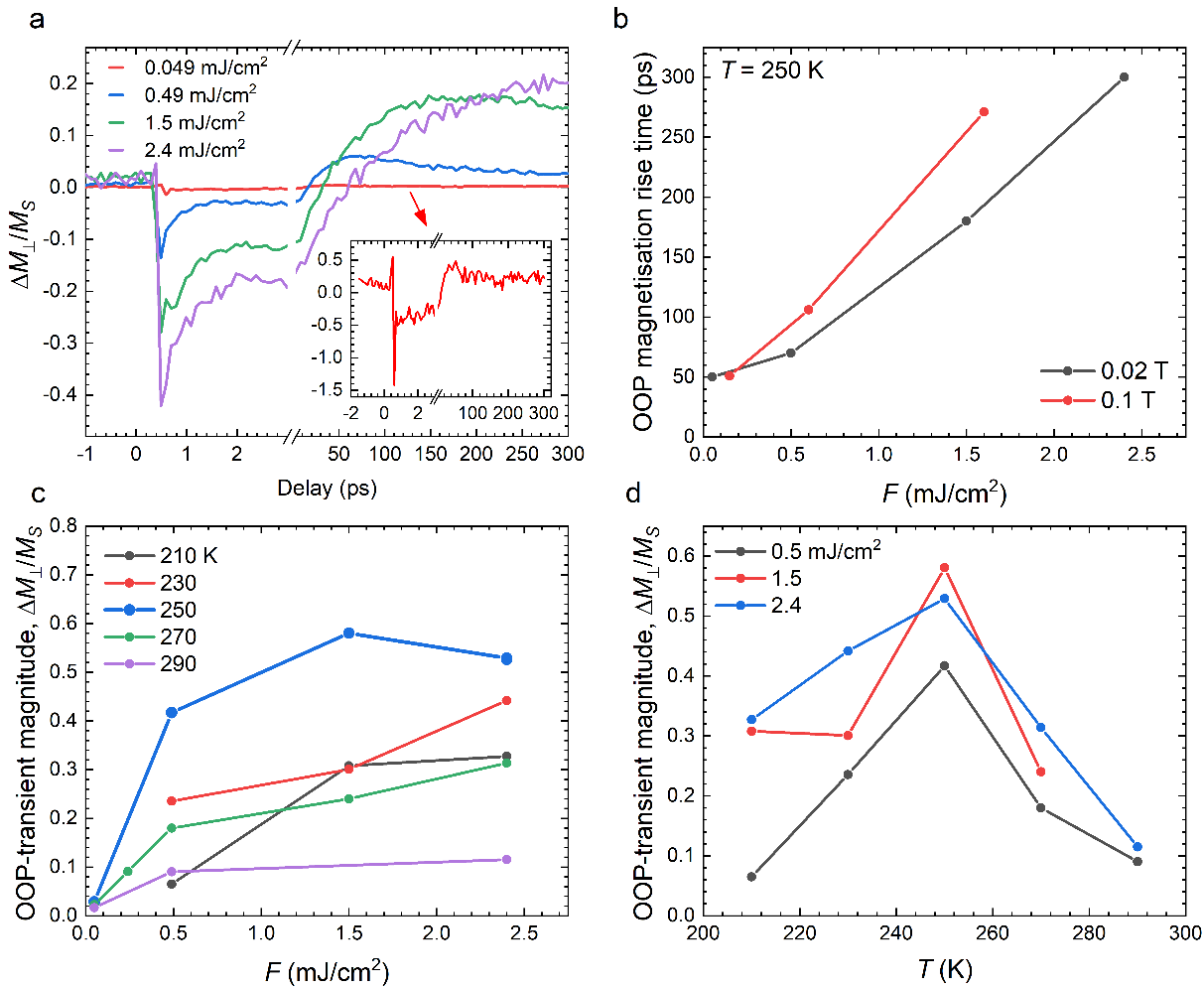


**Figure S23.** Pump-fluence and temperature dependence of the optical-pulse-induced OOP magnetisation transients**. a**, Ratio of onset OOP magnetisation to saturation magnetisation at different fluences at 250K in a weak OOP magnetic field of 0.02 T. **b**, The OOP-magnetisation rise time (the peak transient Kerr angle delay) as a function of the pump fluence at 250K at different OOP magnetic fields of 0.02 T and 0.1 T. **c**, The peak OOP magnetisation swing normalised to the static saturated magnetization (M_S_) as a function of the pump fluence at different temperatures, showing saturation at higher fluences. **d**, The peak OOP magnetisation ($\Delta M_{⟂}$/M_S_) as a function of temperature at different fluences, showing maximal OOP magnetisation swing to be at around 250K in the particular sample, irrespective of the fluence.


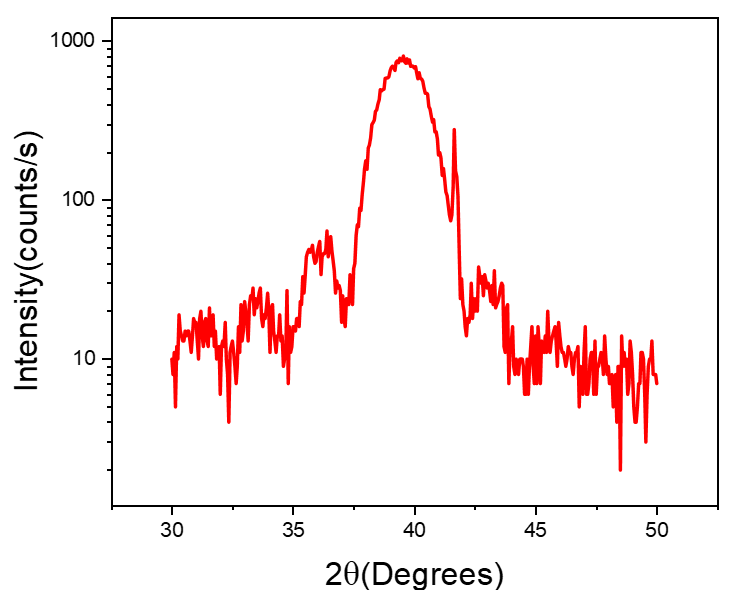


**Figure S24.** Pt(111) X-ray diffraction peak. Pendellosung fringes are visible showing the highly crystalline textured nature of the Pt seed layers.


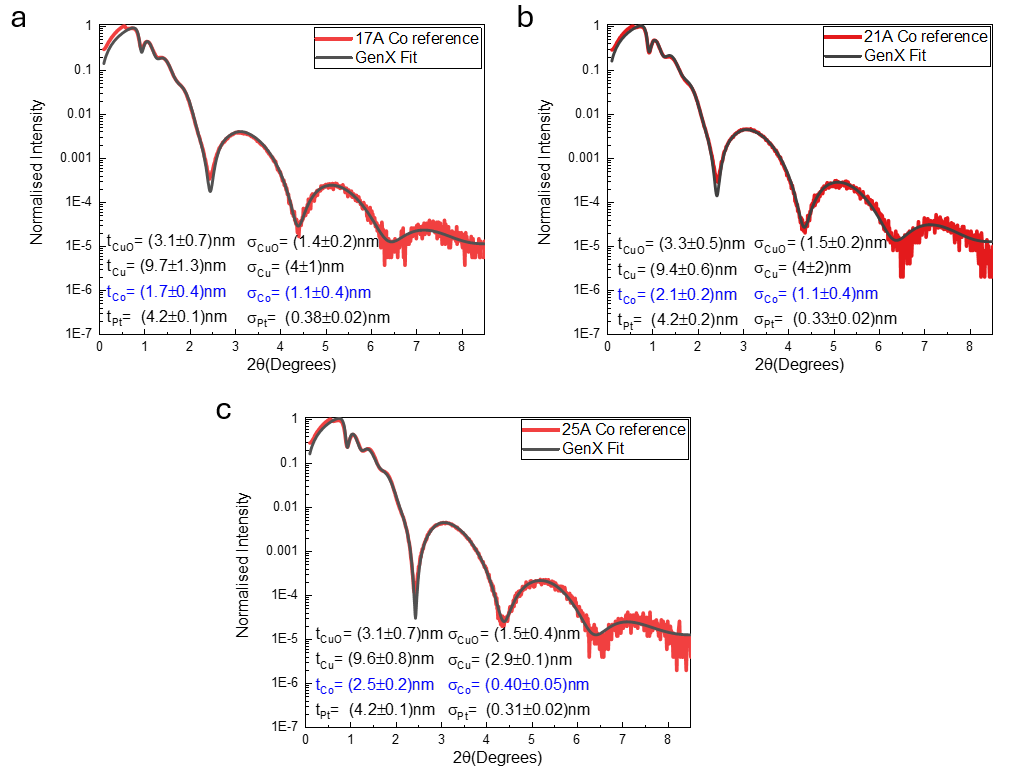


**Figure S25.** X-ray reflectivity characterisation. Reference Cu capped Pt/Co films of varying Co thickness **a**, 1.7nm, **b**, 2.1nm, and **c**, 2.5nm yielding fits in agreement with quartz crystal monitored thicknesses.


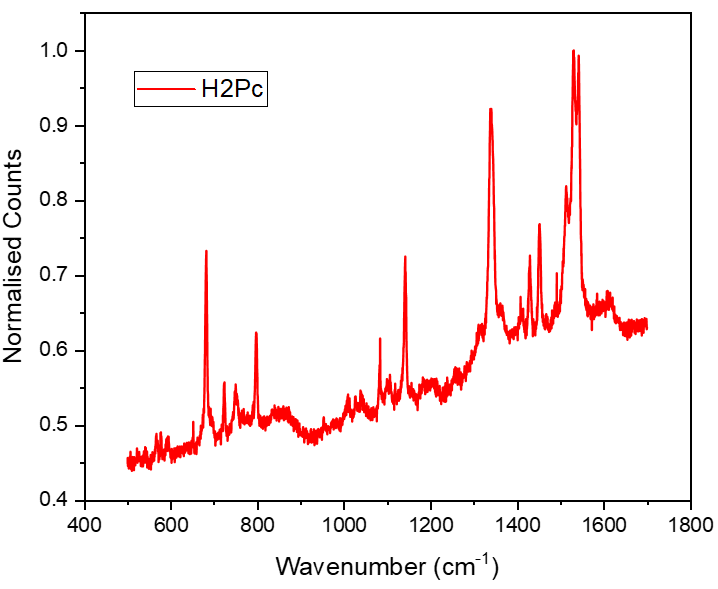


**Figure S26.** Raman spectroscopy of metallo-molecular structure. Characteristic Raman modes of Pc molecules evident on a capped Pt/Co/H_2_Pc structure.
